# The effect of morphology upon electrophysiological responses of retinal ganglion cells: simulation results

**DOI:** 10.1007/s10827-013-0463-7

**Published:** 2013-07-09

**Authors:** Matias I. Maturana, Tatiana Kameneva, Anthony N. Burkitt, Hamish Meffin, David B. Grayden

**Affiliations:** 1 Centre for Neural Engineering, University of Melbourne, 203 Bouverie St, Carlton, Vic 3053 Australia; 2 NICTA Victoria Research Laboratory, Department Electrical & Electronic Engineering, University of Melbourne, Lvl 2, Bld 193, Melbourne, Vic 3010 Australia; 3 Bionics Institute, 384–388 Albert St. East Melbourne, Melbourne, Vic 3002 Australia

**Keywords:** Retinal ganglion cells, Retina, Ion channels physiology, Models, Neurophysiology, Multicompartment modelling

## Abstract

Retinal ganglion cells (RGCs) display differences in their morphology and intrinsic electrophysiology. The goal of this study is to characterize the ionic currents that explain the behavior of ON and OFF RGCs and to explore if all morphological types of RGCs exhibit the phenomena described in electrophysiological data. We extend our previous single compartment cell models of ON and OFF RGCs to more biophysically realistic multicompartment cell models and investigate the effect of cell morphology on intrinsic electrophysiological properties. The membrane dynamics are described using the Hodgkin - Huxley type formalism. A subset of published patch-clamp data from isolated intact mouse retina is used to constrain the model and another subset is used to validate the model. Two hundred morphologically distinct ON and OFF RGCs are simulated with various densities of ionic currents in different morphological neuron compartments. Our model predicts that the differences between ON and OFF cells are explained by the presence of the low voltage activated calcium current in OFF cells and absence of such in ON cells. Our study shows through simulation that particular morphological types of RGCs are capable of exhibiting the full range of phenomena described in recent experiments. Comparisons of outputs from different cells indicate that the RGC morphologies that best describe recent experimental results are ones that have a larger ratio of soma to total surface area.

## Introduction

Photoreceptor cells convert light energy into signals that are transmitted to bipolar cells. Bipolar cells are divided into two types that respond to either increments or decrements in light intensity. Consequently, the signals from photoreceptors are divided into two pathways, the so called ON and OFF pathways. ON and OFF pathways converge at the level of simple cells in the visual cortex.

Retinal ganglion cells (RGCs) are the sole output neurons of the retina. They convert synaptic input from bipolar and other types of inner retinal neurons into signals that carry visual information to the brain. RGCs that respond with increasing spiking frequency to light increments are called ON RGCs. RGCs that increase their spiking frequency with light decrements are called OFF RGCs. OFF-transient cells (OFF T RGCs) are quiet in darkness and have transient spikes at light offset. OFF-sustained cells (OFF S RGCs) fire spontaneous spikes in darkness and have a sustained component of spiking activity during light illumination showed by Murphy and Rieke (2006) and Pang et al. (2003). ON and OFF RGCs can be distinguished not only by their response to light but also by their morphology. In particular, while both ON and OFF RGC somas are located in the retinal ganglion cell layer of the retina, the dendrites of OFF RGCs stratify within sublamina *a* of the inner plexiform layer, while ON cell dendrites stratify within sublamina *b* (Nelson et al. 1978).

Neurons operate in a highly nonlinear manner, generating oscillations and bursting phenomena, thus potentially enhancing the information content of the transmitted signal. RGCs are no exception. It has been shown that OFF RGCs maintain spontaneous activity in the absence of any synaptic input and exhibit subthreshold membrane potential oscillations, rebound excitation and burst firing. On the other hand, ON cells do not show the aforementioned phenomena and require excitatory synaptic input to drive their activity (Margolis and Detwiler 2007).

In an attempt to understand the mechanisms underlying burst firing and subthreshold oscillation in RGCs, a number of studies have examined the voltage-gated channels underlying these phenomena. Rebound excitation is a volley of action potentials at the termination of a period of sustained hyperpolarization. Subthreshold membrane potential oscillations are rhythmic fluctuations in membrane potential that do not result in action potentials. Mechanisms underlying rebound excitation in RGCs were investigated by Mitra and Miller (2007), who showed that low-voltage-activated (LVA) Ca^2+^ and hyperpolarization-activated currents are the main generators of rebound excitation. The availability of LVA Ca^2+^ current in RGCs was also shown by Lee et al. (2003) and Henderson and Miller (2007), while experimental evidence that the hyperpolarization-activated current is present in RGCs was also shown by Lee and Ishida (2007) and Chen and Yang (2007). The role of the persistent sodium current in burst activity was elucidated by van Drongelen et al. (2006) and Traub et al. (2003) who showed the persistent sodium current contributes to fast rhythmic bursting due to its low activation threshold and limited inactivation.

Rebound excitation and subthreshold oscillation were also observed in thalamic neurons (Llinas and Steriade 2006). Similar to RGCs, these phenomena in thalamic neurons were related to LVA Ca^2+^ and hyperpolarization-activated currents. Similarly, it was shown that LVA Ca^2+^ current plays a triggering role in rebound excitation in neurons in the central nervous system (Huguenard 1996). The depolarization of the membrane potential at the termination of a hyperpolarization step opens LVA Ca^2+^ channels producing strong inward Ca^2+^ current that triggers a low threshold calcium spike and a burst of fast and large amplitude sodium action potentials. It was shown that the same current underlies burst generation of thalamocortical relay neurons and plays a central role in the genesis of synchronized oscillations by thalamic cells (Destexhe et al. 1998). In these neurons, the authors showed that LVA Ca^2+^ channels in dendrites must be 4.5-7.6 times higher concentration than in the soma to reproduce experimental results.

Dendritic calcium signaling in ON and OFF RGCs was examined by Margolis et al. (2010). Using simultaneous patch-clamp recordings and two-photon Ca^2+^ imaging, the authors showed pathway-specific differences in voltage-dependent Ca^2+^ signaling. In particular, it was shown that, while both ON and OFF RGCs express high-voltage activated Ca^2+^ current, only OFF cells express LVA Ca^2+^ channels. This result was supported by an earlier study by Guenther et al. (1999), who showed that only a subset of RGCs expressed LVA Ca^2+^ channels.

Computational models provide a formal description of biological processes. Such models serve an important role in testing ideas that are difficult to test experimentally. Computational models can provide detailed realizations of biological systems and processes, suggest experiments in areas that are not well understood, and make specific predictions about cell behaviors. Using compartmental cell models representing simplified RGC morphology, Schiefer and Grill (2006) investigated the effect of an axonal bend on activation threshold, showing that low excitation thresholds near the bend in the axon results in activation of the cells local to the electrode at lower currents than required to excite passing axons. However, the existence of the axonal high density sodium channel band (SOCB) in RGCs was not taken into account in their study. When SOCB properties were taken into account, Jeng et al. (2011) showed that the lowest activation threshold for extracellular electrical stimulation is when the stimulating electrode is placed exactly over the physical location of the SOCB. This computational study agrees well with the experimental results of Fried et al. (2009) who conclude that the action potential initiation site in response to electrical stimulation is in the SOCB. Benison et al. (2001) examined the effect of intracellular calcium diffusion on the amplitude and shape of individual spikes as well as spiking frequency in postnatal cat RGCs. They showed that the diffusion of intracellular Ca^2+^ modeled spatially rather than averaged across the whole cell, was more effective in gating Ca-activated potassium currents. Mechanisms by which cell geometry influences repetitive impulse firing in RGCs were investigated in Fohlmeister and Miller (1997) and found that intercompartmental currents play a major role in determining the impulse spacing and information carried by impulse trains.

Computational modeling was carried out on cell models to explore the effects of ON and OFF RGCs’ morphologies on their intrinsic electrophysiological properties. Although Margolis and Detwiler (2007) explored only mouse *α*-RGCs, we explore 200 cells of different types and morphologies to determine which morphological types can reproduce responses similar to *α*-RGCs. We propose that not all morphological types of RGCs exhibit the phenomena described by Margolis and Detwiler (2007) and show that this is supported by simulations. In this study, we extend our previous single compartment cell model (Kameneva et al. 2011) to more biophysically realistic multicompartment cell models and investigate the effect of cell morphology on intrinsic electrophysiological properties.

## Methods

### Cell morphology

**Fig. 1:**
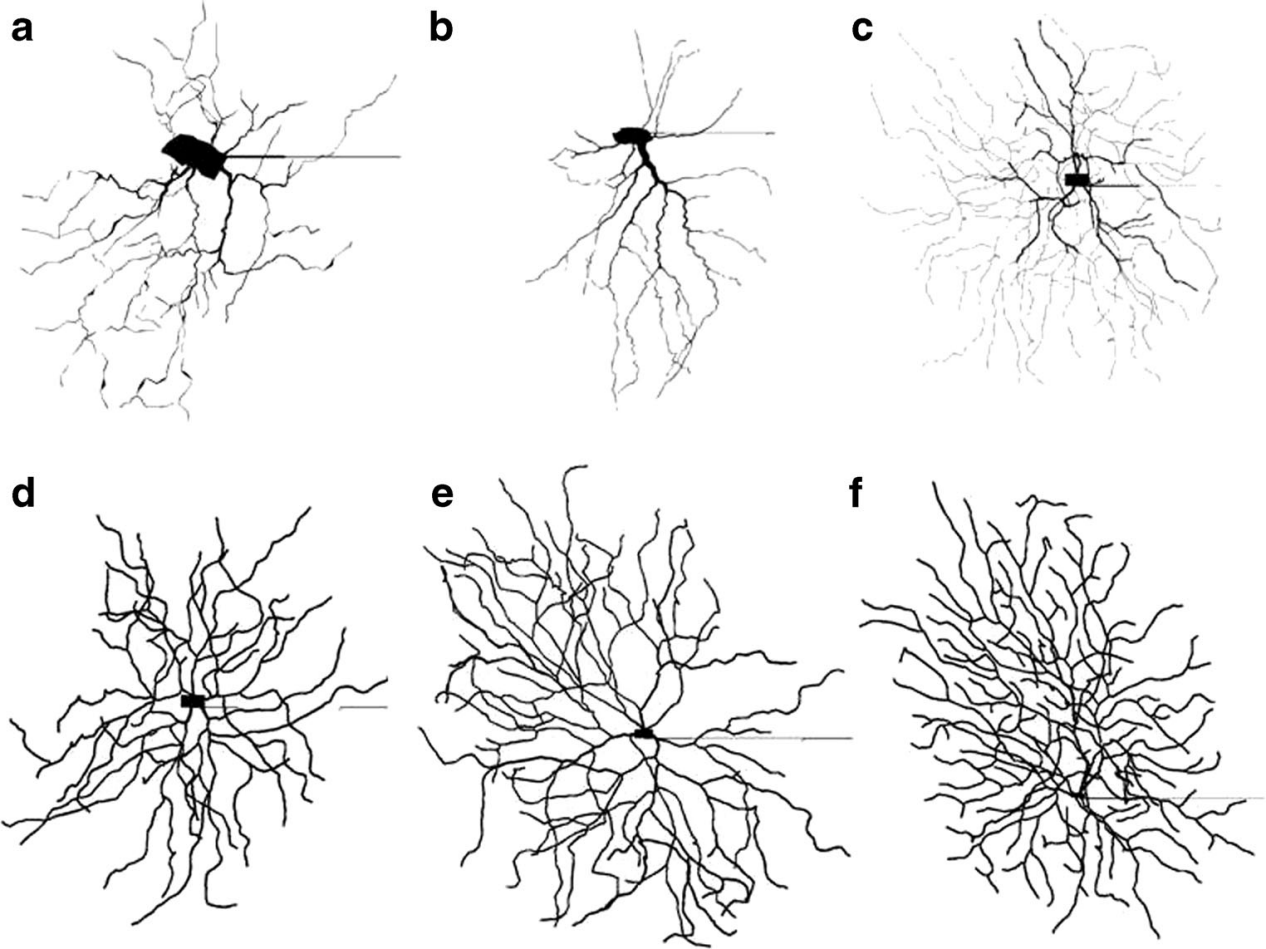
Plots of representative simulated RGC morphologies. Cells show different dendritic structures and soma sizes and shapes. **a**–**c** show cells that met model constraints, while **d**–**f** show cells that did not meet constraints (see Section 2.5). All cell figures were compiled using NEURON (Hines 1993) and adapted from the NeuroMorpho database (Ascoli et al. 2007; Coombs et al. 2006; Toris et al. 1995)

In order to examine the effect of morphology upon the intrinsic electrophysiological response of RGCs, simulations were carried out with two hundred different multicompartment RGC models. Each cell varied in soma and dendrite lengths and diameters, and in dendritic branching structure. Multicompartment RGC structures were taken from the NeuroMorpho database (Ascoli et al. 2007). The cells were divided into two groups: 144 mouse RGCs (Coombs et al. 2006) and 56 salamander RGCs (Toris et al. 1995). Salamander RGCs have long been used in retinal electrophysiological experiments and the *α*-RGCs show similarities in morphology to the mouse A cells (Sun et al. 2002). Although experiments were conducted on mouse cells, simulations using similar types of salamander cells allow the exploration of a larger range of morphologies that can produce *α*-RGC-like behavior. Examples illustrated in Fig. 1 are created using NEURON (Hines 1993). The cells in Fig. 1a–c show cell models that have a large soma and dendrites that have decreasing diameters. Figure 1d–f show cell models that have small to medium sized soma and generally uniform diameter dendrites. While model tuning and validation were based on mouse A type RGCs, it is unclear what the morphological types of the mouse and salamander cells are in the NeuroMorpho database. Most likely they contain a variety of cells from different morphological classes.

**Fig. 2:**
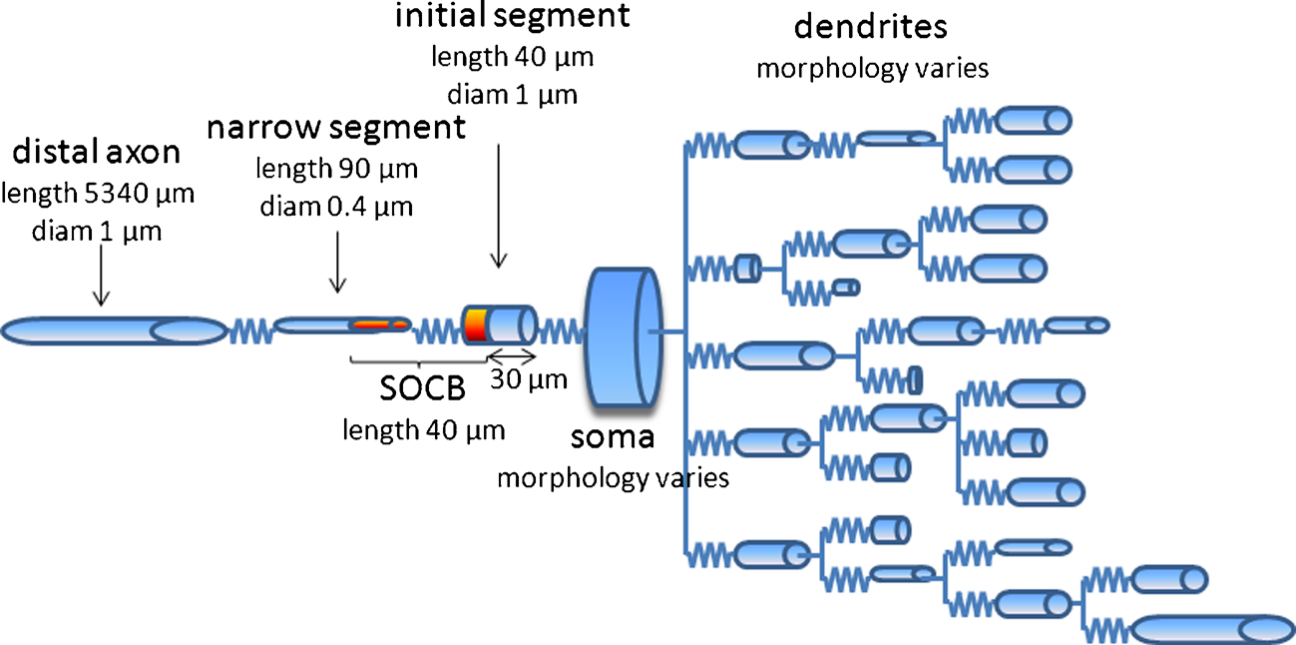
A schematic diagram of the cylinder model of a cell’s morphology used in simulations. Compartments are represented by cylindrical elements. Soma and dendrite morphology (influencing the cylinders’ lengths and diameters) varied from cell to cell. The morphology of the axon was the same for all cells

The cell models were divided into compartments representing the dendrites, soma, and axon as illustrated in Fig. 2. In all cells, the axon comprised four regions: the initial segment, sodium channel band (SOCB), narrow segment, and distal axon. The initial segment refers to the proximal portion of the axon that stems from the soma. This segment was of uniform diameter equal to the diameter of the distal axon. The narrow segment connects to the initial segment 40 *μ*m from the soma and extends for 90 *μ*m. The narrow segment was modeled as a cylinder with uniform diameter of 0.4 *μ*m, consistent with proportions used by Toris et al. (1995). The SOCB refers to a region comprising the distal part of the initial segment and the proximal part of the narrow region. This region was located 30 *μ*m proximal to the soma and extended 40 *μ*m. This region had a higher concentration of sodium and persistent sodium channels. The distal axon had a diameter of 1 *μ*m and a fixed length of 5340 *μ*m for all cells. This is consistent with dimensions used in all salamander cells (Toris et al. 1995). The morphologies of the mouse cells in the NeuroMorpho database did not include an axon or included only a very short axon. In order to monitor action potential propagation in the axon and to allow consistency between the cell types, the mouse morphologies were modified to include the four axonal compartments described above.

The morphological properties of the cells are summarized in Table 1. All cell models have unique morphologies apart from the axon. For all cells, the total surface area, *S*_total_, soma surface area, *S*_soma_, axon surface area, *S*_axon_, axon diameter, *D*_axon_, and average dendrite diameter, *D*_dendrite_, were calculated for comparison. The ratios of dendrite to total surface area, *R*_dend,total_, and soma to total surface area, *R*_soma_, were also computed. In addition, the number of dendritic tips (end of a dendritic branch), number of dendritic bifurcations, and average dendritic compartment lengths were also recorded. Cell surfaces areas were calculated by summing the surface area of the individual cylindrical compartments, omitting the end faces. In order to differentiate the unique cell properties, the total surface area excluded the axon.

**Table 1:** Explored cells morphological statistics (200 cells in total). Cells taken from the NeuroMorpho database (Ascoli et al. 2007). Shown are the cell’s minimum (Min), maximum (Max), average (Ave) and standard deviation (St Dev) values

	*S*_total_ Total surface area	*S*_soma_ Soma surface area	*S*_axon_ Axon surface area	*S*_dendrite_ Dendrite surface area	*D*_axon_ Axon diameter	*D*_dendrite_ Average dendrite diameter
	*μ*m^2^	*μ*m^2^	*μ*m^2^	*μ*m^2^	*μ*m	*μ*m
Min	969	10	17020	292	1	0.09
Max	32457	2196	17020	31773	1	2.18
Ave	9099	488	17020	8613	1	0.78
St Dev	6004	425	0	6089	0	0.37

	Number of dendritic tips	*R*_dend,total_ Dendrite to total surface area ratio	*R*_soma,total_ Soma to total surface area ratio	Number of bifurcations	Average dendritic compartment length *μ*m	
Min	7	0.30	0.0005	13	8	
Max	377	0.99	0.70	754	142	
Ave	62	0.90	0.10	123	42	
St Dev	44	0.14	0.14	88	26	

### Model parameters

Cells responses were simulated in the NEURON environment (Hines 1993). The membrane properties were described using Hodgkin-Huxley type equations and included leak (*I*_L_), sodium (*I*_Na_), calcium (*I*_Ca_), delayed rectifier potassium (*I*_K_), A-type (*I*_K,A_), and Ca-activated potassium (*I*_Ca_) currents. The conductances of these currents remained fixed during simulations. T-type low voltage activated (LVA) calcium (*I*_T_), hyperpolarization-activated (*I*_h_), and sodium persistent (*I*_NaP_) currents were also introduced and allowed to vary to explore the effects of these currents on the cells’ responses. Simulations involved systematic parameter searches for the maximal ionic conductances of sodium persistent, *ḡ*_NaP_, T-type LVA calcium, *ḡ*_T_, and hyperpolarization-activated, *ḡ*_h_, conductances satisfying experimental data.

The RGC ionic currents were summed using Kirchoff’s law,

1 $$\begin{array}{rll} C_{\mathrm{m}}\frac{dV}{dt} &=&\bar{g}_{\mathrm{L}}(V-V_{\mathrm{L}})+\bar{g}_{\mathrm{Na}}m^3h(V-V_{\mathrm{Na}})\\ &&+\bar{g}_{\mathrm{Ca}}c^3(V-V_{\mathrm{Ca}})+(\bar{g}_{\mathrm{K}}n^4+\bar{g}_{\mathrm{K,A}}a^3h_{\mathrm{A}} \\ &&+\bar{g}_{\mathrm{K(Ca)}})(V-V_{\mathrm{K}})+\bar{g}_{\mathrm{h}}l(V-V_{\mathrm{h}})+\bar{g}_{\mathrm{T}}m_{\mathrm{T}}^3h_{\mathrm{T}} \\ &&\times(V-V_{\mathrm{T}})+\bar{g}_{\mathrm{NaP}}p(V-V_{\mathrm{Na}})+I_{\mathrm{stim}},\\ \end{array} $$

where *V * is the membrane potential, *C*_m_ is the specific capacitance of the membrane, and *ḡ* is the maximum conductance of the ionic current defined by the subscript. Leak, sodium, calcium, delayed rectifier potassium, A-type, and Ca-activated potassium currents have dynamics as described by Fohlmeister and Miller (1997). The hyperpolarization-activated, T-type LVA calcium, and sodium persistent currents were modeled as by van Welie et al. (2006) and Wang et al. (1991) and Traub et al. (2003), respectively. *I*_stim_ is an intracellular stimulation current. Since the data of Margolis and Detwiler (2007) was obtained under synaptic blockage, we did not include synaptic currents in the model.

In contrast to sodium and potassium ions, the normal intracellular calcium ion concentration is so low that it can be increased dramatically during a single depolarization response. At rest, the cytoplasmic free calcium level is between 20 and 300 nM. Since calcium replenishment operates by a different mechanism than other ions, involving intracellular calcium storage rather than transmembrane ion pumps, the calcium reversal potential varied with time according to

2 $$V_{\mathrm{Ca}}(t)=\frac{RT}{2F} \; \ln \left( \frac{\left[\mathrm{Ca}^{2+}\right]_{\mathrm{e}}}{\left[\mathrm{Ca}^{2+}\right]_{\mathrm{i}}(t)} \right), $$

where *R* is the gas constant, *T* is temperature in Kelvin, *F* is the Faraday constant, and [Ca^2+^]_e_ represents the extracellular calcium ion concentration in normal Ringer’s solution. The intracellular calcium ion concentration [Ca^2+^]_i_ varied in response to *I*_Ca_ (the calcium current, the third term on the right of Eq. (1) according to Fohlmeister and Miller (1997)

3 $$\frac{d\left[\mathrm{Ca}^{2+}\right]_{\mathrm{i}}(t)}{dt}=\frac{-3I_{\mathrm{Ca}}(t)}{2F r} -\frac{\left[\mathrm{Ca}^{2+}\right]_{\mathrm{i}}(t)-\left[\mathrm{Ca}^{2+}\right]_{\mathrm{res}}}{\tau_{\mathrm{Ca}}},$$

where *r*=0.1*μ*m is the depth of the shell beneath the membrane for the calcium pump and *τ*_Ca_=1.5 ms is the time constant for the calcium current. The free intracellular calcium ions above the residual level, [Ca^2+^]_res_=0.1*μ*M, are removed from the cell. At rest, *V*Ca is approximately 120 mV.

The conductance of the Ca-activated potassium channels was modeled as

4 $$\bar{g}_{\mathrm{K(Ca)}}(t)= g_{\mathrm{K(Ca)}} \cdot \frac{\left(\left[\mathrm{Ca}^{2+}\right]_{\mathrm{i}}(t)/\left(\mathrm{Ca}^{2+}\right)_{\mathrm{diss}}\right)^2} {1+\left(\left[\mathrm{Ca}^{2+}\right]_{\mathrm{i}}(t)/\left[\mathrm{Ca}^{2+}\right]_{\mathrm{diss}}\right)^2},$$

where *g*_K(Ca)_ = 5·10^−5^ S/cm^2^ and [Ca^2+^]_diss_ = 10^−6^ is the calcium dissociation constant.

The numerical values for the parameters used in simulations are given in Table 2. The experimental data used to constrain and validate our model was obtained at 30–34 °C. Therefore, simulations were run at 32 °C. The gating variables $m, h, c, n, a, h_{\mathrm {A}}, l, m_{\mathrm {T}}, p$ of the voltage-gated ion channels operate according to the first-order kinetic equation

5 $$\frac{dx}{dt}=-(\alpha_{x} + \beta_{x})x + \alpha_{x},$$

where *x* is a gating variable and indexes the appropriate rate constant $\alpha _{x}$ and $\beta _{x}$. The inactivation gating variable, *h*_T_, for the *I*_T_ current was modeled as having two closed states (Wang et al. 1991). The rate of change of *h*_T_ had the second-order dynamics,

6 $$\dot{h}_{\mathrm{T}}=\alpha_{h_{\mathrm{T}}}(1-h_{\mathrm{T}}-d)-\beta_{h_{\mathrm{T}}}h_{\mathrm{T}},$$

where *d* satisfies

7 $$\dot{d}=\beta_{d}(1-h_{\mathrm{T}}-d)-\alpha_{d}d.$$

**Table 2:** Simulation parameters

Temperature	T=32 °C
Membrane capacitance	$C_{m}=1 \; \mu $ F/cm^2^
Potassium reversal potential	*V*_K_ = −70 mV
Leak reversal potential	*V*_L_ = −60 mV
Hyperpolarization-activated reversal potential	*V*_h_ = 0 mV
LVA calcium reversal potential	*V*_T_ = 120 mV
Sodium reversal potential	*V*_Na_ = 35 mV
Calcium reversal potential	*V*_Ca_ ≈ 120 mV at rest; is variable, see (2)
Calcium dissociation constant	[Ca^2+^]_diss_ = 10^−6^ M
Extracellular calcium ion concentration	[Ca^2+^]_e_ = 1.8 mM
Gas constant	*R* = 8.314 J/(M.K)
Faraday constant	*F* = 9.684 · 10^4^ C/M

The expressions for the voltage-dependent rate constants are given in Table 3.

**Table 3:** Rate constants for voltage-gated ion channels. (V is measured in mV)

Na^+^ channel	$\alpha _{m} = \frac {-0.6 (V+30)}{\mathrm {exp}(-0.1(V+30))-1}$	$\beta _{m} = 20 \mathrm {exp}\left (\frac {-(V+55)}{18}\right )$
	$\alpha _{h} = 0.4 \mathrm {exp}\left (\frac {-(V+50)}{20}\right )$	$\beta _{h} = \frac {6}{1 + \mathrm {exp}(-0.1(V+20))}$
Ca^2+^ channel	$\alpha _{c} =\frac {-0.3(V+13)}{\mathrm {exp}(-0.1(V+13))-1}$	$ \beta _{c} = 10 \mathrm {exp}\left (\frac {-(V + 38)}{18}\right )$
K^+^ channel	$\alpha _{n} = \frac {-0.02 (V+40)}{\mathrm {exp}(-0.1(V+40))-1}$	$\beta _{n} = 0.4 \mathrm {exp}\left (\frac {-(V + 50)}{80}\right )$
A channel	$\alpha _{a} = \frac {-0.006 (V+90)}{\mathrm {exp}(-0.1(V+90))-1}$	$\beta _{a} = 0.1 \mathrm {exp}\left (\frac {-(V + 30)}{10}\right )$
	$\alpha _{h_{\mathrm {A}}} = 0.04 \mathrm {exp}\left (\frac {-(V+70)}{20}\right )$	$\beta _{h_{\mathrm {A}}} =\frac { 0.6}{1 + \mathrm {exp}(-0.1 (V+40))}$
h channel	$\alpha _{l} = \mathrm {exp}(0.08316(V+75))$	$\beta _l =\mathrm {exp}(0.033264(V+75))$
T channel	$\alpha _{m_{\mathrm {T}}}= \left (1.7 + \mathrm {exp}\left ( \frac {-(V + 28.8)}{13.5} \right )\right )^{-1}$	$\beta _{m_{\mathrm {T}}}= \frac {1 + \mathrm {exp}({-(V +63)}{7.8})}{1.7 +\mathrm {exp}\left (\frac { -(V + 28.8)}{13.5}\right )}$
	$\alpha _{h_{\mathrm {T}}} = \mathrm {exp}\left (\frac {-(V +160.3)}{17.8}\right )$	$\beta _{h_{T}}=\alpha _{h_{T}}\left [0.25 + \mathrm {exp}\left (\frac {V + 83.5}{6.3}\right )\right ]^{0.5}-0.5\alpha _{h_{T}}$
	$\alpha _{d} =\frac {1 + \mathrm {exp}\left (\frac {V + 37.4}{30}\right )}{240 \left [0.5 + \left (0.25 + \mathrm {exp}\left (\frac {V + 83.5}{6.3}\right )\right )^{0.5}\right ]} $	$\beta _{d} = \alpha _{d}\left [0.25 +\mathrm {exp}\left (\frac {V + 83.5}{6.3}\right )\right ]^{0.5}$
NaP channel		
[V<−40]	$\alpha _{p} = \frac {0.025 + 0.14 \mathrm {exp}\left (\frac { V + 40 }{ 10}\right )}{1 + \mathrm {exp}\left (\frac { - (V+48 ) }{ 10} \right )}$	$\beta _{p}=\frac { 1-\left (1 + \mathrm {exp}\left ( \frac { - (V+48 ) }{ 10} \right )\right )^{-1}}{0.025 + 0.14 \mathrm {exp}\left ( \frac { V + 40 }{ 10}\right )} $
[V≥−40	$\alpha _{p} = \frac {0.02 + 0.145 \mathrm {exp}\left ( \frac {-(V+ 40) }{ 10}\right )}{1 + \mathrm {exp}\left ( \frac { - (V+48)}{10} \right )}$	$\beta _{p}=\frac {1-\left (1 + \mathrm {exp}\left ( \frac { - (V+48 )}{10}\right )\right )^{-1}}{0.02 + 0.145 \mathrm {exp}\left ( \frac {-(V +40)}{10}\right )}$

*Ion channel distributions*

The different concentrations of ion channels that were used in different compartments of each RGC are given in Table 4. *ḡ*_NaP_, *ḡ*_T_, and *ḡ*_h_ were allowed to vary from [10^−15^, 10^−1^] S/cm^2^. $\bar {g}_{\mathrm {h}}$ varied with the same concentration in all regions. The leak current was increased due to the three additional inward currents in comparison to Sheasby and Fohlmeister model as described earlier.

**Table 4:** Distribution of ionic channels in cells compartments. Conductances above the dashed line were constant across all simulations while conductances below were allowed to vary. Dash indicates the absence of such conductance in the compartment. $\bar {g}_{\mathrm {L}_0}$=8×10^−6^ S/cm^2^ represents the original leak value taken from Fohlmeister and Miller (1997). All conductances are in units of S/cm^2^

	Soma	Dendrites	Initial segment	Narrow region	SOCB	Axon
*ḡ*_Na_	**0.08**	**0.025**	**0.15**	**0.2**	**5 × 0.08**	**0.07**
*ḡ*_Ca_	**0.0015**	**0.002**	**0.0015**	−	−	−
*ḡ*_K_	**0.018**	**0.012**	**0.018**	**0.018**	−	**0.018**
*ḡ*_K,A_	**0.054**	**0.036**	**0.054**	−	−	−
*ḡ*_K(Ca)_	**0.000065**	**0.000001**	**0.000065**	**0.000065**	−	0.07
*ḡ*_L_	**15** × $ {\bar{\mathbf{g}}}_{\mathbf{L_0}}$	**15** × $ {\bar{\mathbf{g}}}_{\mathbf{L}_0}$	**15** × $ {\bar{\mathbf{g}}}_{\mathbf{L}_0}$	**15** × $ {\bar{\mathbf{g}}}_{\mathbf{L}_0}$	**15** × $ {\bar{\mathbf{g}}}_{\mathbf{L}_0}$	**25** × $ {\bar{\mathbf{g}}}_{\mathrm{L}_0}$
*ḡ*_h_	*ḡ*_h_	*ḡ*_h_	*ḡ*_h_	*ḡ*_h_	−	*ḡ*_h_
*ḡ*_NaP_	*ḡ*_NaP_	*ḡ*_NaP_	0.05 × *ḡ*_NaP_	0.05 × *ḡ*_NaP_	5 × *ḡ*_NaP_	0.05 × *ḡ*_NaP_
*ḡ*_T_	*ḡ*_T_	5 × *ḡ*_T_	*ḡ*_T_	*ḡ*_T_	*ḡ*_T_	*ḡ*_T_

Previous RGCs models (Fohlmeister and Miller 1997; Sheasby and Fohlmeister 1999) have found that in order to reproduce experimentally observed phenomena, it was necessary to increase the sodium channel density in the initial axonal region proximal to the soma. This is supported by recent evidence showing the existence of a low threshold region consisting of a dense band of voltage-gated sodium channels in the initial portion of the RGC axon (Fried et al. 2009; Jeng et al. 2011). This band is located 38.8 ±10 *μ*m from the soma, with a mean length of 36.8 ± 5.6 *μ*m. The exact concentration of sodium in the SOCB in RGCs is unclear.

We modeled the SOCB region with dimensions described in Section 2.1 and with a *ḡ*_Na_ and *ḡ*_NaP_ SOCB to soma ratio, *R*_SOCB,soma_, of five. This is consistent with (Jeng et al. 2011) who modeled the SOCB having an increased *ḡ*_Na_ ratio of 5–50 times the soma concentration. Using this, we found that a distal axon *ḡ*_NaP_ 20 times smaller than the soma *ḡ*_NaP_ was necessary in order to ensure orthodromic propagation of action potentials and prevent spontaneous activity being generated in the distal axon when there was no spontaneous activity in the soma.

*Adjusting leak conductance*

In comparison to the Sheasby and Fohlmeister (1999) model, an increased leak conductance was necessary in our model due to three additional inward currents, *I*_NaP_, *I*_h_ and *I*_T_, that were not present in the Sheasby and Fohlmeister model. The modification was required to compensate for the additional ion channels without modifying the conductances of other channels. When the maximal leak conductance, *ḡ*_L_, was initially set uniformly throughout the cell to 8×10^−6^ S/cm^2^, similar to previous models (Sheasby and Fohlmeister 1999), high frequency spontaneous activity was produced even with low levels of *ḡ*_NaP_ and *ḡ*_NaP_, and also action potentials were evoked during a hyperpolarizing current injection. This value of *ḡ*_L_ also resulted in the resting potential being 10–30 mV lower than experimentally recorded (Margolis and Detwiler 2007). In addition, using the value of *ḡ*_L_ used by Sheasby and Fohlmeister (1999) resulted in the membrane potential during a hyperpolarizing current injection falling more than 100 mV below the resting potential. This is 3–4 times lower than the potential reached in plots shown in Margolis and Detwiler (2007), which show that the minimum membrane potential reached during a hyperpolarizing current injection was around 30 mV below the resting potential. In order to find an appropriate leak conductance, we investigated the effect of the leak conductance on the cell’s input resistance and membrane potential during a hyperpolarizing current injection.

### Model tuning

A subset of experimental data from Margolis and Detwiler (2007) was used to evaluate the model parameters *ḡ*_NaP_, *ḡ*_T_, and *ḡ*_h_. The following data were used for model tuning:

- The value of the mean resting potential for ON cells and the mean resting potential for OFF cells.
- The frequency of the spontaneous activity in OFF sustained (OFF S) and OFF transient (OFF T) cells and the absence of spontaneous activity in ON cells.
- The absence of action potentials during a hyperpolarizing step of −0.2 nA in both ON and OFF cells.
- Presence (in OFF cells) or absence (in ON cells) of high frequency burst firing at the termination of a hyperpolarizing step of −0.2 nA.

A comparison of simulation parameter constraints and experimental data is given in Table 5. The resting potential for spontaneously spiking cells was calculated as by Margolis and Detwiler (2007) by removing spikes 2 ms after reaching a 10 V/s threshold and calculating an average over at least 1 s. To calculate the cell’s spiking frequency, only action potentials that overshot 0 mV were counted; we call this constraint C0. The timing of a spike was calculated by recording the time the soma potential exceeded a threshold of 0 mV. This timing information was then used to calculate a cell’s spiking frequency. Burst frequency was calculated over 200 ms after termination of the hyperpolarizing current step.

**Table 5:** Comparison of simulation parameter constraints and experimental data

Constraint	ON RGCs	Model Constraints	Experimental data
C0		Spikes overshoot 0 mV	
C1 ON	resting potential	[−62, −70] mV	−65.5± 1.3 mV
C2 ON	Spontaneous Frequency	0 Hz	0 Hz
C3 ON	Spike rate during 500 ms stimulus of −0.2 nA	0 Hz	0 Hz
C4 ON	Burst rate at the termination of 500 ms stimulus of −0.2 nA	0 Hz	0 Hz
	OFF RGCs	Model Constraints	Experimental data
C0		Spikes overshoot 0 mV	
C1 OFF	resting potential	[−50, −62] mV	−54.6 ± 1.0 mV (OFF T) −55.1± 1.1 mV (OFF S)
C2 OFF	Spontaneous Frequency	[15, 23] Hz (OFF T)	20.3 ± 3.0 Hz (OFF T)
		[40, 48] Hz (OFF S)	43.3 ± 3.5 Hz (OFF S)
C3 OFF	Spike rate during 500 ms stimulus of −0.2 nA	0 Hz	0 Hz
C4 OFF	Burst rate at the termination of 500 ms stimulus of −0.2 nA	Rebound excitation ≥ 2 × spontaneous frequency	Rebound excitation 60 Hz

The constraints labeled C1 ON, C1 OFF, and C2 OFF are less stringent than the narrow ranges observed in the experimental data allowing the exploration of a larger range of RGCs morphologies. C4 ON further constrained the parameter space by requiring absence of burst firing at the termination of a negative current step for ON cells. In C4 OFF, the spiking rate during rebound excitation was calculated based on a plot of a sample cell from Margolis and Detwiler (2007) and, therefore, should be taken as indicative only. Frequency has been calculated over 200 ms after the termination of a hyperpolarizing step.

A search of the parameter space for *ḡ*_NaP_, *ḡ*_T_, and *ḡ*_h_ was undertaken using variable iteration steps. The parameter space explored was in the range *ḡ*_NaP_, *ḡ*_T_, *ḡ*_h_ ∈ [10^−15^, 1] S/cm^2^. Initially, logarithmic steps of 10 S/cm^2^ were used to explore the parameter space. In order to obtain higher resolution of the ON and OFF sets, smaller regions were explored where *ḡ*_h_ varied with logarithmic steps of 10, and *ḡ*_NaP_ and *ḡ*_T_ varied with linear steps. A large parameter space was explored since we expected to find multiple conductance combinations satisfying the constraints listed above. Points satisfying the constraints listed above were recorded and used to determine the conductance limits in *ḡ*_NaP_, *ḡ*_T_, *ḡ*_h_ producing *α*-RGC-like behavior. Access to the high performance computational facilities at the Victorian Life Science Computation Initiative (VLSCI) allowed exploration of a large number of morphologies. Parallel simulations using 50 processors were employed to obtain a total of 75,000 CPU hours worth of simulated data.

### Model validation

The ability of the model to generalize was tested by comparing the model outputs to data (Mitra and Miller 2007; Margolis and Detwiler 2007) that were not used to constrain the model. Validation criteria below refer only to OFF RGCs since ON cells do not show these phenomena in experiments. Choosing validation criteria that were not used to constrain the model tested the model’s predictive capability.

Comparisons of the validation data (labeled V1-V3) and model outputs were made as follows:

- V1: Subthreshold oscillations in OFF cells. When a hyperpolarizing current is applied, OFF cells should reveal oscillations in the subthreshold regime with a rate of 1–10 Hz, similar to the data of Margolis and Detwiler (2007).
- V2: Disruption of spontaneous activity reveals bursting behavior in OFF cells. With negative current steps of increasing amplitude, OFF cells should exhibit a transition from regular spontaneous activity, to irregular activity, to burst firing, and then to silence, as shown experimentally by Margolis and Detwiler (2007).
- V3: Coefficient of variation of the inter-spike interval in OFF cells. The coefficient of variation (CV) is defined as the standard deviation of the inter-spike interval divided by the mean. It is a common measure of the irregularity of spiking, in which a high value CV reflects high variability and a low value of CV reflects high regularity. The CV of the inter-spike interval (ISI) in OFF cells should increase with increasing level of hyperpolarization (Margolis and Detwiler 2007).

To check for V2 and V3, 10 cells that met constraints C0–C4 were simulated and the CV was plotted against the membrane potential. To calculate the mean membrane potential, spikes were first removed by calculating the derivative of the membrane potential, and removing 2 ms of data after a 10 mV/ms threshold was reached. The membrane potential was calculated as the mean potential after spikes were removed. The CV was calculated in response to increasing −20 pA current steps, to a maximum of −80 pA.

## Results

To investigate the effect that cell morphology has on the ability to produce the experimentally recorded phenomena listed in Table 5, we compared the total surface areas of the cells and the dendritic surface areas of the cells that met the constraints with those that did not. Furthermore, we explored the effect of reduction of the soma and dendrite surface area on the intrinsic electrophysiological properties of the cells.

In addition to the distribution of ionic channels listed in Table 4, other distribution were explored where the concentration of *ḡ*_NaP_ and *ḡ*_T_ were varied in different parts of the cell. Most model variations were eliminated due to unphysiological behaviors described in Section 3.1 or lack of the model’s ability to describe *α*-like behavior.

### Cell morphology

**Fig. 3:**
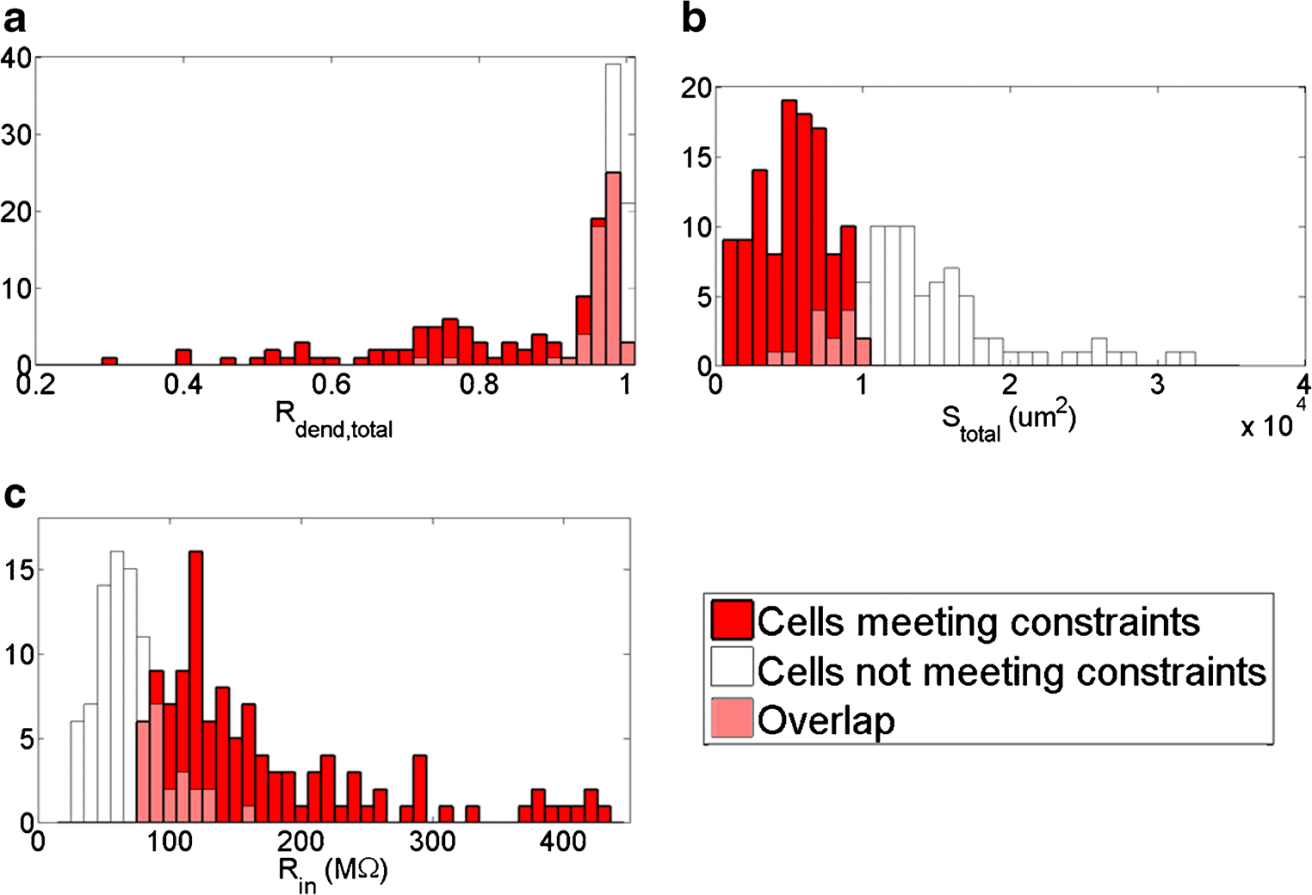
Histograms comparing cells that meet model constraints C0-C4 (*red*) and cells that do not (*white*). The overlay is shown in pink. **a** Ratio of dendritic to total surface area, *R*_dend,total_. **b** Total surface area (*μ*m^2^), *S*_total_. **c** Input resistance into the dendrites from the soma, *R*_in_. Means and standard deviations given in Table 6

To examine how the total surface area of the cells affects the intrinsic electrophysiology, we computed the total surface area, *S*_total_, and the ratio of the dendritic surface area to the total surface area, *R*_dend,total_. We compared these morphological features with cells that could meet model constraints C0–C4 from Table 5, and cells that could not meet the constraints. From a total of 200 simulated cells, 88 cells did not meet constraints C0–C4. One third of these did not meet constraint C0 because they failed to produce action potentials that reached a spike threshold of 0 mV. The remaining two-thirds of cells that did not meet the constraints, could not for a variety of reasons including spiking during hyperpolarizing stimulus (C3 ON and C3 OFF). These cells also tended to be cells with larger *S*_total_, larger *R*_dend,total_ and a lower input resistance, *R*_in_. Table 6 shows a comparison of cells meeting constraints C0–C4, and those not meeting constraints. Figure 3 shows histograms depicting how cells were distributed according to *S*_total_, *R*_dend,total_ and *R*_in_.

**Table 6:** Comparison of cells meeting constraints C0–C4 and those not

	Cells meeting constraints		Cells not meeting constraints	
	Mean	Standard deviation	Mean	Standard deviation
*R*_dend,total_	0.84	0.16	0.97	0.04
*S*_total_ (*μ*m^2^)	5230	2380	14226	5494
*R*_in_ (MΩ)	172.8	88	68.7	25

The effect of morphology on electrophysiology was investigated further by systematically reducing the cell’s dendritic and soma surface areas, while keeping ion channel densities constant. A subset of mouse cells were simulated with the original morphology, then each dendritic compartment diameter was systematically reduced by 10 % to 50 % in 10 % steps. The same protocol was used to explore the effect of reduction of the soma surface area. The resting potential, the derivative of the membrane potential, and spontaneous and rebound burst frequencies were calculated for each reduction in area.

**Fig. 4:**
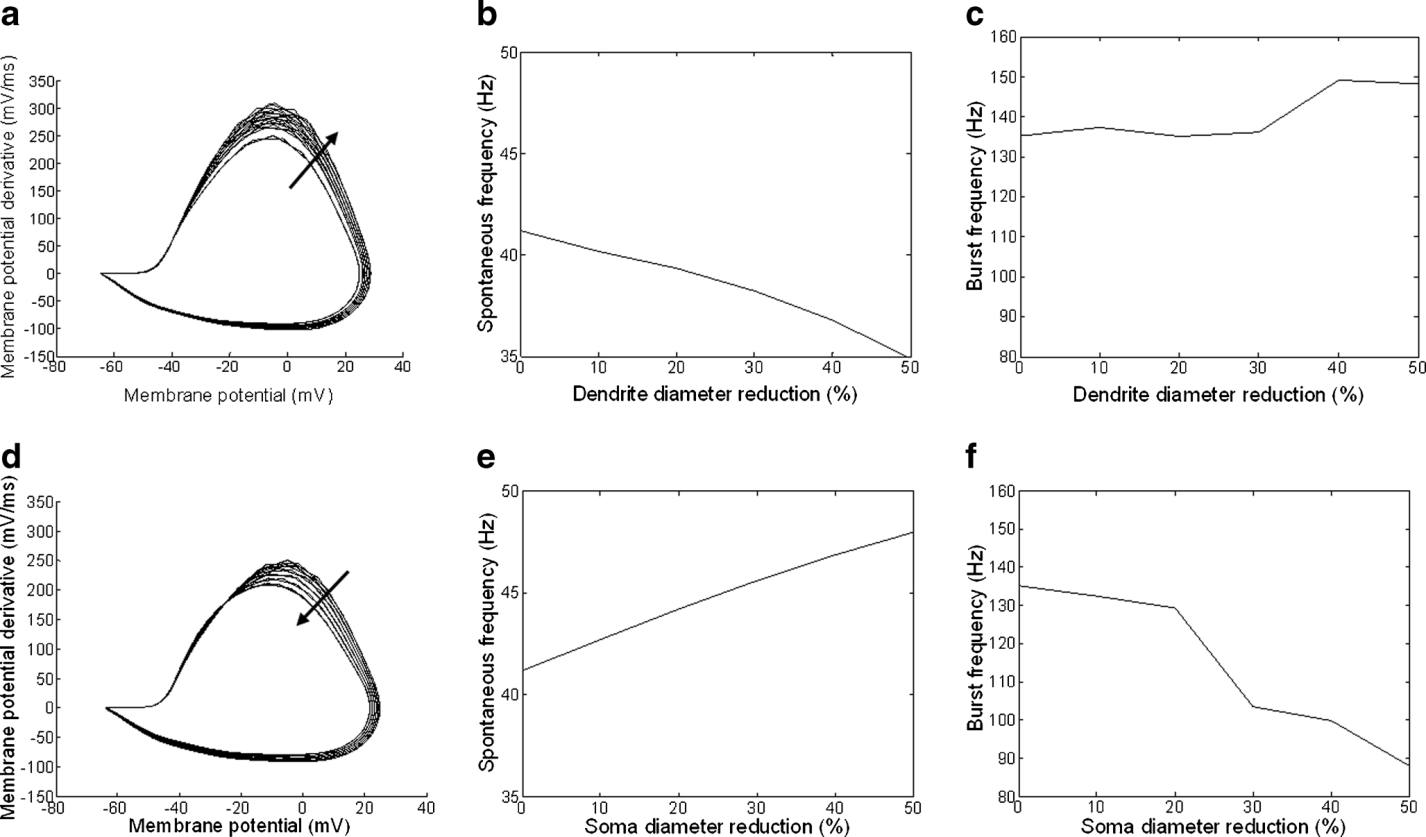
Effect of reducing the dendritic surface area and of reducing the soma’s surface area on a cell’s responses. **a**–**c** An OFF S cell was simulated under normal conditions and then with the diameter of each dendritic compartment reduced by 10–50% in steps of 10%. **a** Phase plot showing that as the surface area is decreased (*arrow*), the action potentials increase in maximal amplitude and reach a higher rate of change. **b** The spike frequency shows a decrease in response to the change in morphology. **c** The burst frequency shows an increasing trend in response to the change in morphology. **d**–**f** An OFF S cell was simulated under normal conditions and then with the diameter and length of the soma reduced by 10–50 % in steps of 10 %. **d** Phase plot showing that as the surface area is decreased (*arrow*), the action potentials decrease in maximal amplitude and reach a lower maximal rate of change. This results in slower action potentials reaching lower amplitudes. **e** The spike frequency shows a steady increase in response to the change in morphology. **f** The burst frequency shows a decreasing trend in response to the change in morphology. In simulations *ḡ*_NaP_ = 3.33 × 10^−6^ S/cm^2^, *ḡ*_T_ = 5.36 × 10^−4^ S/cm^2^, and *ḡ*_h_ = 10^−9^ S/cm^2^

Responses of an OFF S cell with dendritic and soma surface area reduction are shown in Fig. 4. The phase plot of the membrane potential for this cell is shown in Fig. 4a and d. The arrows show the direction of the phase plot change with decreasing dendritic and soma diameters. Dendritic and soma surface area reduction had significant effects on the phase plot shape and on the cells’ spontaneous and burst frequencies. In particular, the phase plot shows that as the dendritic surface area was decreased, the action potentials increased in maximal amplitude and reached a higher rate of change. This resulted in faster action potentials reaching higher amplitudes. Also, the spontaneous frequency decreased with decreasing dendritic diameter but the burst frequency increased (see Fig.4b,c). The opposite happened when the soma surface area was decreased (see Fig. 4e,f). Note that although the surface area was reduced in simulations, the ionic channel conductance concentrations remained fixed.

The cells were also simulated with a reduction in surface area by reducing each dendritic compartment length from 10 % to 50 % in 10 % steps. The results were similar to the results when the dendrite diameters were decreased.

Cells that did not meet the constraints tended to show single action potentials with small voltage fluctuations in the soma. These fluctuations in the membrane potential generally emerged from cells that had very large *S*_total_. Having a larger *S*_total_ meant that a larger depolarizing potential was necessary to bring the cell to spiking threshold, thereby requiring more than 1 depolarization emerging from the SOCB to cause an action potential. These cells also needed larger hyperpolarizing current steps in order to silence their spontaneous activity. Morphologically, these cells tended to have uniform dendrites with large diameters. Conversely, cells meeting constraints tended to have a mixture of thick and thin dendrites and tapered off much more significantly than cells not meeting constraints C0-C4. A representative sample of cells meeting the constraints are shown in Fig. 1a–c. Figure 1d–f shows representative cells that did not meet the constraints. Note the differences in the morphology between cells in (a–c) and cells in (d–f), in particular the smaller somas and uniform diameter dendrites in (d–f) compared to (a–c).

### Model parameters

*Adjusting leak conductance*

The maximal leak conductance, *ḡ*_L_, was initially set uniformly throughout the cell to 8 × 10^−6^ S/cm^2^, similar to previous models (Sheasby and Fohlmeister 1999). Increasing *ḡ*_L_ had the effect of increasing the resting potential, and decreasing *R*_in_.

**Fig. 5:**
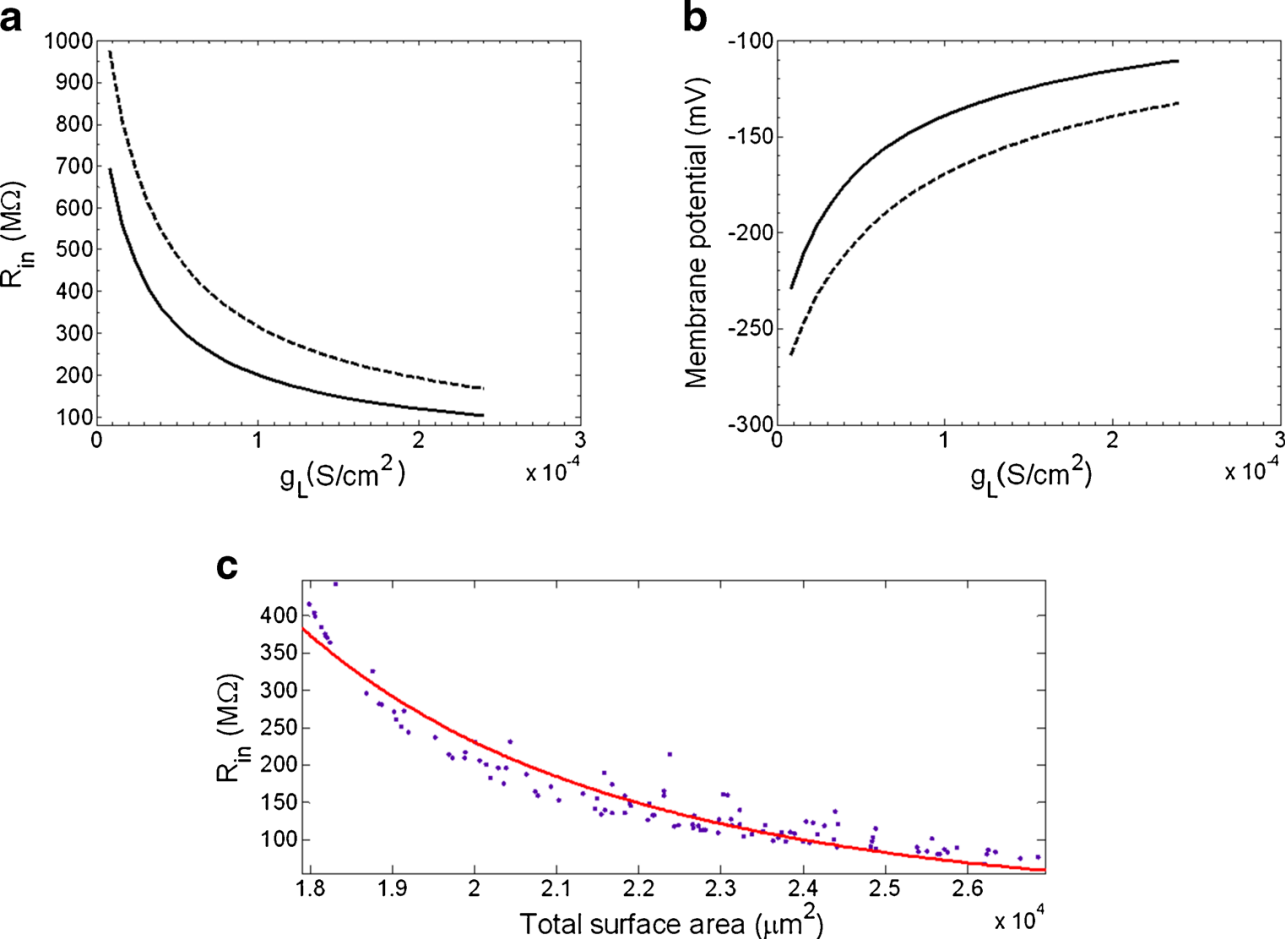
Effect of leak conductance, *ḡ*_L_, and total surface area, *S*_total_, on the input resistance, and on the membrane potential during a −0.2 nA current step. **a** Input resistance, *R*_in_ as a function of *ḡ*_L_. **b** Membrane potential during a −0.2 nA current step as a function of *ḡ*_L_. In simulations for **a** and **b**, *dashed*: cell from the Salamander set; *Solid*: cell from the mouse set. In simulations, *ḡ*_NaP_ = 1 × 10^−5^ S/cm^2^, *ḡ*_T_ = 3 × 10^−4^ S/cm^2^ and = 1 × 10^−9^ S/cm^2^. **c** Input resistance, *R*_in_ as a function of total surface area, *S*_total_. Each individual dot represents a cell. *Red*: curve fit using a power fit of the form *y*=*ax* ^b^, where *a* = 1.17 × 10^22^,*b* = −4.58. In simulations *ḡ*_NaP_ = 1 × 10^−5^ S/cm^2^, *ḡ*_T_ = 3 × 10^−4^ S/cm^2^, *ḡ*_h_ = 1 × 10^−9^ S/cm^2^, and *ḡ*_L_ = 1.2 × 10^−4^ S/cm^2^

In order to find an appropriate value of the *ḡ*_L_, cells were simulated with increasing levels of *ḡ*_L_. *R*_in_ as a function of *ḡ*_L_ for two cells is given in Fig. 5a. Cells were simulated firing spontaneously for 1000 ms; *R*_in_ was calculated over the last 500 ms of the simulations. Input resistance into the dendrites was calculated by taking the average resistance of each dendrite stemming from the soma. Simulations showed that *R*_in_ decreased exponentially as *ḡ*_L_ increased. When *ḡ*_L_ was set to 8 × 10^−6^ S/cm^2^, the cell’s input resistance was positioned at a very high level, much higher than values reported in more recent studies (O’Brien et al. 2002; Margolis and Detwiler 2007). By increasing the leak conductance to 1.2 × 10^−4^ S/cm^2^ (15 fold increase), the resting potential was increased by 10–20 mV to around −60 mV. This value of the resting potential was closer to values reported in previous studies (O’Brien et al. 2002; Margolis and Detwiler 2007) for OFF RGCs. This also decreased the *R*_in_ to approximately one fifth of its previous value. Further increasing *ḡ*_L_ did not significantly reduce *R*_in_ since the relationship between *R*_in_ and *ḡ*_L_ was exponential. For this reason, an increase of 15 times was used uniformly throughout the cell, except in the distal axon, where it was increased by 25 times. This positioned the resting potential to potentials closer to the level defined in constraints C1 ON and C1 OFF.

Increasing *ḡ*_L_ also had the effect of raising the membrane potential during a −0.2 nA current step. Figure 5b shows how the membrane potential during a −0.2 nA current step changed as *ḡ*_L_ was increased. For some cells, the increase in membrane potential over the range shown in Fig. 5b was as high as 300 mV. For most cells, an increase in *ḡ*_L_ to *ḡ*_L_ = 1.2 × 10^−4^ S/cm^2^ positioned the membrane potential during a − 0.2 nA current step at around −100 mV. This is consistent with hyperpolarization potentials shown in various figures by Margolis and Detwiler (2007).

*R*_in_ varied substantially from cell to cell. The mean *R*_in_ for cells meeting all constraints C0-C4 was 172 MΩ with a standard deviation of 88 MΩ. This is comparable to values measured by Margolis and Detwiler (2007), where OFF S cells were recorded between 100 and 140 MΩ, OFF T cells around 60 MΩ, and ON cells around 55 MΩ. Figure 5c shows how the cell’s input resistance varies in relationship to the total surface area of the cell. The simulation shows a trend of decreasing *R*_in_ with increased cell surface area. This is consistent with (O’Brien et al. 2002) who show that alpha cells, which have the largest surface area, have the lowest input resistance, and zeta cells, which have the smallest surface area, have the largest input resistance.

**Fig. 6:**
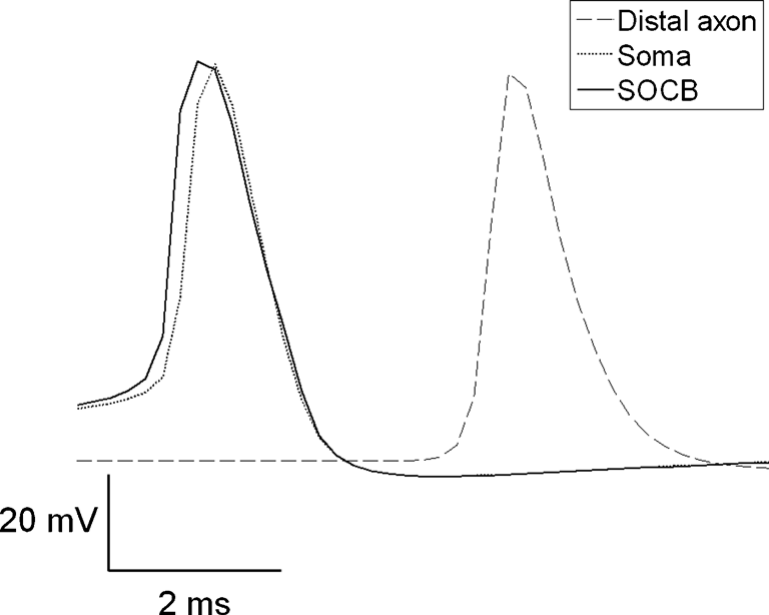
The action potential initiation site is in the axon’s SOCB. Voltage traces are shown for a representative cell. Recordings are made in the soma, distal axon and SOCB. For this simulation *ḡ*_NaP_ = 1 × 10^−5^ S/cm^2^, *ḡ*_T_ = 3 × 10^−4^ S/cm^2^ and *ḡ*_h_ = 1 × 10^−9^ S/cm^2^

Simulations showed that a higher conductance of *I*_NaP_ is necessary in the axonal region corresponding to the SOCB, while a lower *ḡ*_NaP_ is necessary in the rest of the axon. This ensured that the action potential initiation site was always in the SOCB. A representative example of a recorded action potential in the soma, dendrite, and axon is given in Fig. 6. The time of the action potential peak in the axon segment corresponding to the SOCB is 0.2 ms earlier than it’s time in the soma and 2 ms earlier than in the distal axon.

**Fig. 7:**
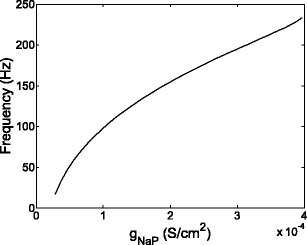
Spiking frequency as a function of *ḡ*_NaP_ is plotted for a representative cell, showing a monotonically increasing relationship between *ḡ*_NaP_ and spontaneous frequency. For this simulation *ḡ*_T_ = 3 × 10^−4^ S/cm^2^ and *ḡ*_h_ = 1 × 10^−9^ S/cm^2^

An almost linear relationship between spontaneous frequency and *ḡ*_NaP_ was found. Results showed that values of *ḡ*_NaP_ above 2 × 10^−5^S/cm^2^ led to spontaneous activity, as illustrated in Fig. 7. The *ḡ*_NaP_ activation threshold varied across cells but remained within 1×10^−5^−3×10^−5^ S/cm^2^. Note that an increase in *ḡ*_T_ also led to an increase in spontaneous activity, generally requiring *ḡ*_T_ greater than 3 × 10^−4^ S/cm^2^ to activate the cell when *ḡ*_NaP_ was removed; however, the cell output failed to meet constraint C2 ON and OFF, and C3 ON and OFF.

### Model tuning

Using the constraints listed in Table 5, we found distinct sets of the parameters (*ḡ*_NaP_, *ḡ*_T_, *ḡ*_h_) that correspond to ON, OFF T, and OFF S cell populations. The parameter sets for ON, OFF T and OFF S RGCs are mutually exclusive. Valid sets were defined as connected conductance sets produced by cells meeting constraints outlined in Table 5. Note that other ionic conductance distributions explored contained cells that could produce points satisfying constraints C0-C4; however, these were eliminated due to the inability to produce valid sets that contained connected conductance sets.

**Fig. 8:**
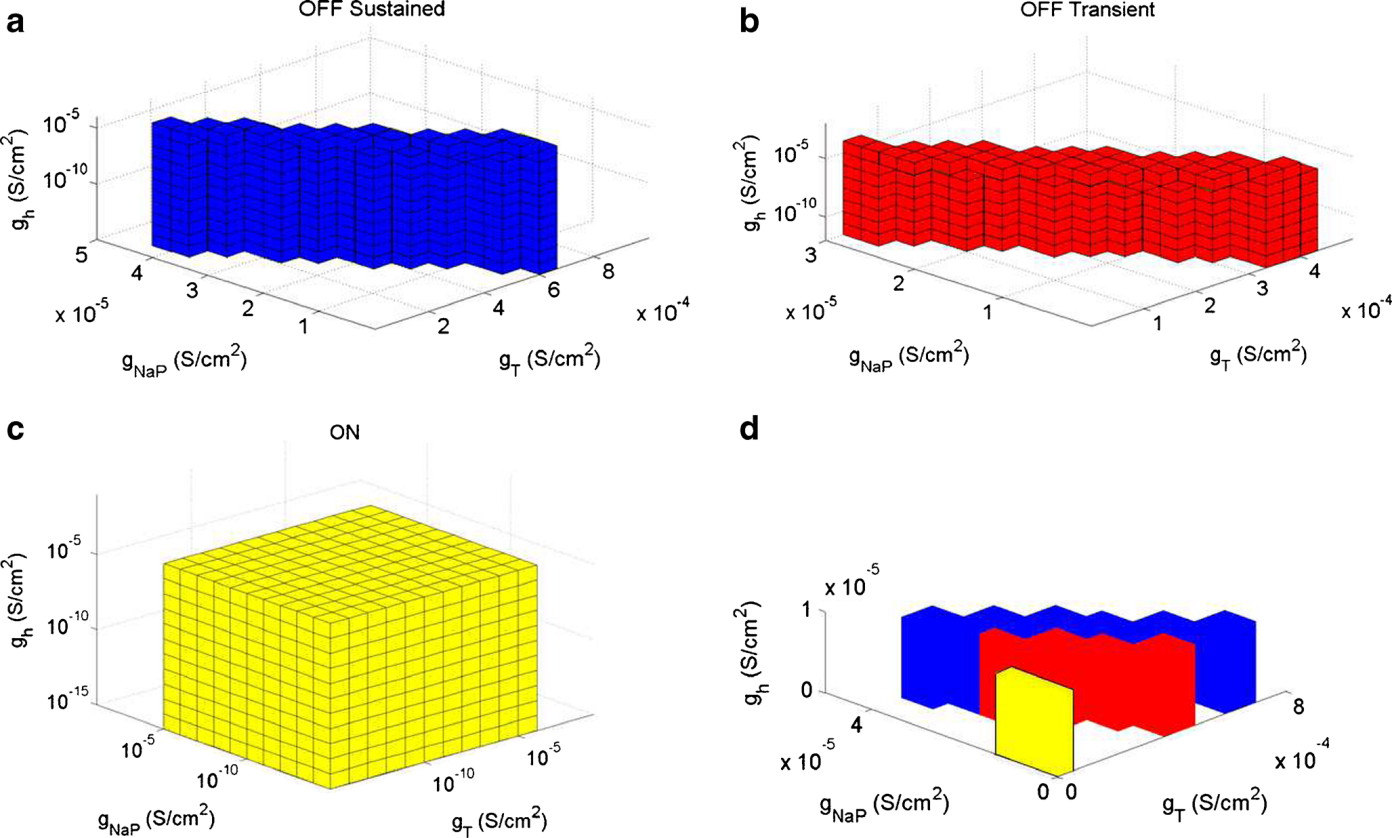
Typical ON and OFF RGCs parameter sets for conductances *ḡ*_h_, and *ḡ*_T_. Colored cubes indicate parameter values that satisfy the constraints C0 – C4. **a** OFF S RGCs set. **b** OFF T RGCs set. **c** ON RGCs set. **d** Representative figure to give an indication of how each set fits together within the parameter space. No sets overlap. Logarithmic steps were used to explore the parameter space in *ḡ*_h_ while linear steps were used for *ḡ*_T_ and *ḡ*_NaP_ in OFF RGCs. Logarithmic steps were used in ON cells. The sets were found under model constraints C0 – C4 ON for the ON set and C0 – C4 OFF for the OFF sets (see Table 5). The cell illustrated here was a mouse cell. Results are typical for all cells producing valid sets. All parameters in the Hodgkin-Huxley scheme were fixed except for *ḡ*_h_, *ḡ*_T_ and *ḡ*_NaP_. The parameter space *ḡ*_h_ ∈ [10^−15^, 1] S/cm^2^, *ḡ*_T_ ∈ [10^−15^, 1] S/cm^2^, *ḡ*_NaP_ ∈ [10^−15^, 1] S/cm^2^ was explored. However, a reduced parameter space is illustrated to emphasize the region producing valid sets

A total of 112 cells (54 of the Salamander cells, and 58 of the Mice cells) produced valid sets. An example of the conductance sets for a cell is illustrated in Fig. 8. The parameter space shown was typical for all cells producing valid sets; however, the linear dependence between *ḡ*_T_ and *ḡ*_NaP_ differed slightly for each cell. Other model variations were able to show cells producing sets similar to those shown in Fig. 8, however, the number of cells were much less. Also, the sets produced tended to be much smaller than those presented in Fig. 8, often contained protrusions, and were unable to show the validating constraints like subthreshold oscillations.

In general, the ON set constraints C0 ON – C4 ON (refer to Table 5) were satisfied for the conductances in the following ranges: *ḡ*_NaP_ ≤ 10^−5^, *ḡ*_h_ ≤ 10^−5^, and *ḡ*_T_ ≤ 10^−5^ S/cm^2^. The set did not contain any protrusions or troughs. The set was essentially a rectangular prism indicating that the constraints on *ḡ*_NaP_, *ḡ*_T_, and *ḡ*_h_ were satisfied independently of one another.

The OFF-T set constraints C0 OFF – C4 OFF were satisfied in the approximate ranges *ḡ*_NaP_ ≤  3 10^−5^, *ḡ*_T_ ∈ [3 × 10^−5^, 4 × 10^−4^], and *ḡ*_h_ ≤ 10^−5^ S/cm^2^.

The OFF-S set constraints C0 OFF – C4 OFF were satisfied in the ranges similar to OFF T: *ḡ*_NaP_ ≤ 3.5 × 10^−5^, *ḡ*_T_ ∈ [3.5 × 10^−5^, 7 × 10^−4^], and *ḡ*_h_ ≤ 10^−5^ S/cm^2^.

The parameter set region was found to be roughly the same for all morphologies of RGCs that satisfied constraints C0 – C4. However, the shape of the parameter set region varied slightly between cells within the limits given above.

All sets showed little or no dependence on *ḡ*_h_. These results and the validation results show that while *I*_h_ produces the characteristic sag seen at the onset of a negative current injection, it only has a modulatory role in shaping the cell response.

### Model validation

**Fig. 9:**
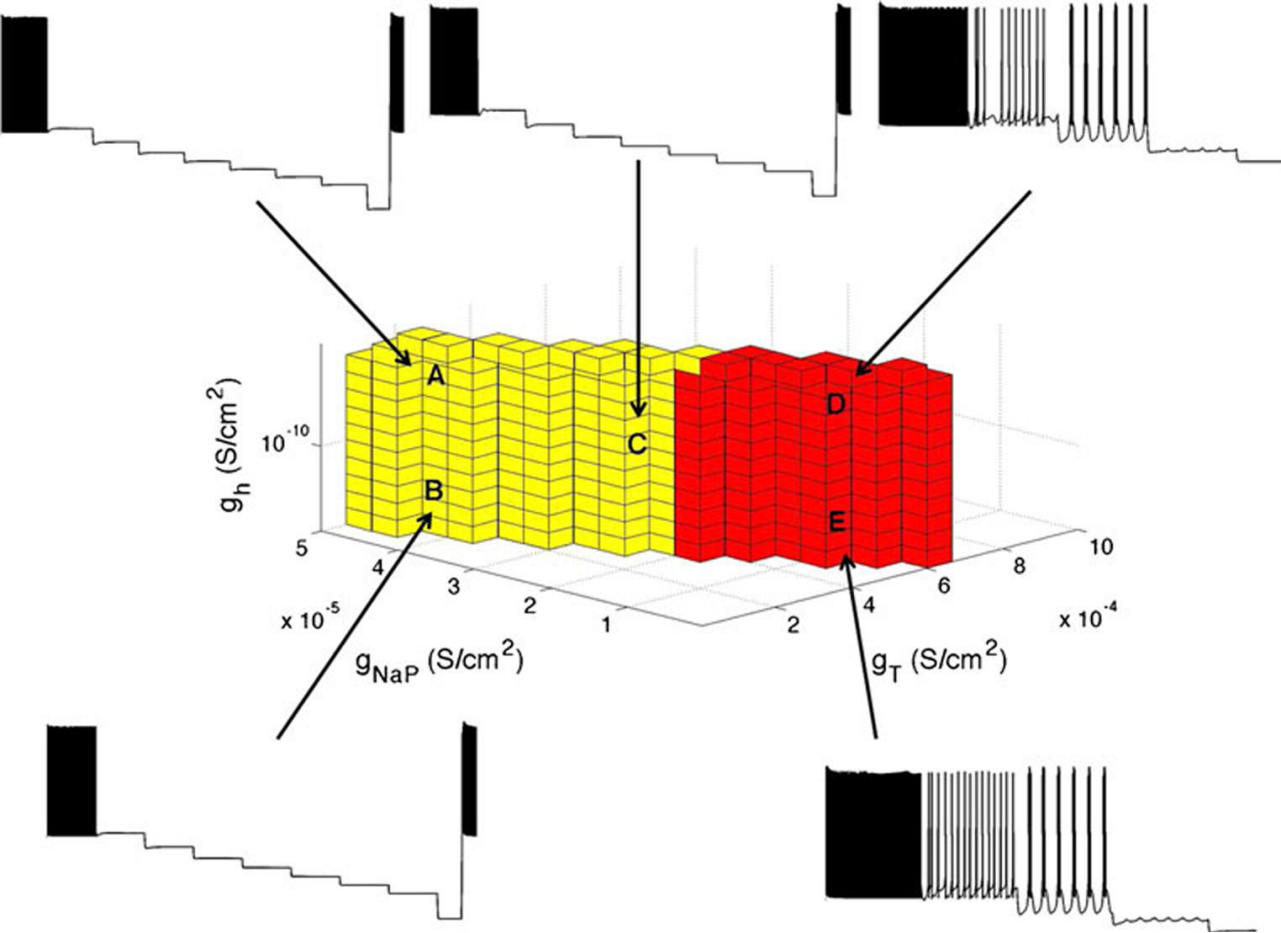
The ability of the cells to produce subthreshold oscillations and burst firing for conductance points A, B, C, D, and E is illustrated. Shown are voltage traces for five points from a mouse cell. Voltage traces for all five points are in response to seven increasing hyperpolarizing current steps, each of −0.02 nA and each for 2 s duration. The eighth current step is −0.2 nA and 0.5 s duration. Voltage traces for points D and E are cropped to highlight the cell’s response. For this cell, only conductances in the red region were able to produce sustained oscillations

To validate the model, outputs were compared to experimental data V1 – V3 that were not used to constrain the model. In order to explore the ability of the cells to produce subthreshold oscillations and burst firing, a sample of 80 cells was chosen. Five conductance points in each set with conductance values denoted by A, B, C, D, and E in Fig. 9 were chosen to check for the presence of oscillations and burst firing. These points changed slightly from cell to cell according to the conductance set produced for each cell. A total of eight current steps each of 2 s duration was simulated for a total of 17 s.

Figure 9 gives an example of the cell’s response to a hyperpolarizing step current. Shown are voltage traces taken for the five points for a mouse cell. Only points D and E were able to produce sustained oscillations. While this is typical of most of the cells producing oscillations, some cells also produced oscillations at point C. This indicates that high levels of *ḡ*_T_ are necessary in order to produce oscillations. Note that the inserted voltage traces are illustrative, and the time and voltage axis may be different for each trace.

Figure 9 shows that the interplay between *ḡ*_NaP_ and *ḡ*_T_ is crucial for generating subthreshold oscillations. *I*_L_ also plays an important role in positioning the membrane potential in the activation range of *I*_NaP_ and playing a modulatory role in subthreshold oscillations. With a low amplitude hyperpolarizing current injection, cells are hyperpolarized to approximately −63 mV, close to the reversal potential for *ḡ*_L_. The presence of *I*_NaP_ provides the constant inward current that depolarizes the membrane potential and creates a feedback loop with *ḡ*_L_. In response, *ḡ*_L_ oscillates from an inward to outward current. These oscillations are soon damped if *ḡ*_T_ is only present in small concentrations. With higher concentrations of *ḡ*_T_, small and rapid membrane depolarization activate large spikes of *I*_T_ current. This provides the necessary drive to create fluctuations in the membrane potential and burst of spikes at a higher level of hyperpolarization. Maximal levels of *ḡ*_T_ resulted in sustained oscillations of anywhere between 0.5 and 10 mV in amplitude.

The presence of subthreshold oscillations was checked in 80 out of the 112 cells that produced valid sets. The presence of subthreshold oscillations varied substantially across different cell morphologies. 37 cells out of 80 cells were able to show these features in at least one of C, D, or F. In all cases, only points C, D, or E in Fig. 9 could satisfy *V*1 and *V*2 indicating that *ḡ*_T_ had to be greater than 3 × 10^−4^ S/cm^2^ to show the phenomenon. In all cases, only conductances from the OFF S set could produce sustained oscillations. Conductances from the OFF T set could only produce damped oscillations.

An example of the proportion of the points in the set satisfying validation constraint *V*1 for an OFF S cell is given by the red cubes in Fig. 9. The proportion was typical for OFF S cells that produced subthreshold oscillations. The amplitude of oscillation was 1–5 mV, the frequency of oscillation was between 1 and 10 Hz, which corresponds well with experimental results of 2–8 Hz from Margolis and Detwiler (2007). Parameter values represented by cubes in red produced sustained oscillations. Some of the parameter values corresponding to yellow cubes close to the red section produced damped oscillations.

**Fig. 10:**
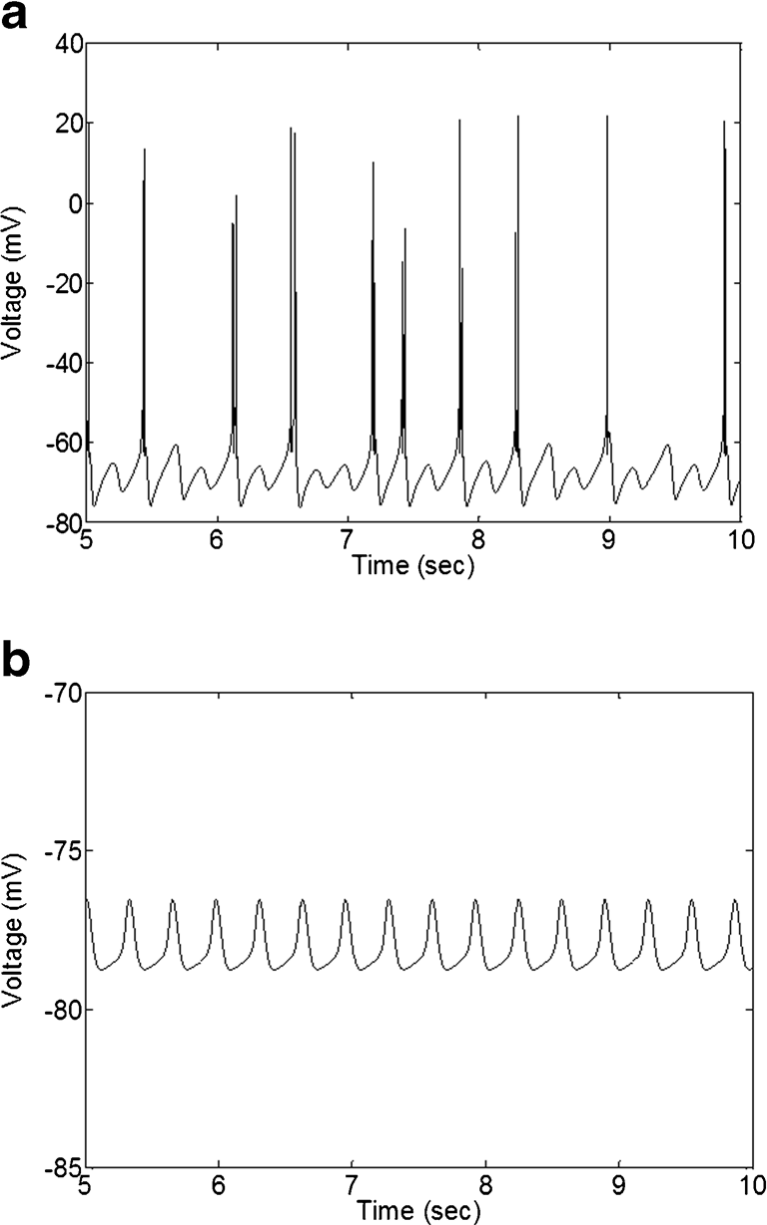
Responses of two points from the red set in Fig. 9 to a hyperpolarizing current step. Both plots show oscillations between 3 and 5 Hz. **a** *ḡ*_NaP_ = 6.67 × 10^−6^ S/cm^2^, *ḡ*_T_ = 4.69 × 10^−4^ S/cm^2^, *ḡ*_h_ = 10^−5^ S/cm^2^, and I_*stim*_ = −0.07 nA. **b** *ḡ*_NaP_ = 6.67 × 10^−6^ S/cm^2^, *ḡ*_T_ = 5.36 × 10^−4^ S/cm^2^, *ḡ*_h_ = 10^−14^ S/cm^2^, and I_*stim*_ = −0.06 nA

Figure 10 shows responses from a mouse cell of two points from the red region in Fig. 9 to a hyperpolarizing current step. Figure 10a shows oscillations with bursting behavior. This is similar to the type of oscillations OFF T cells produced in our simulations. Figure 10b shows subthreshold oscillations for an OFF S cell. Oscillations were observed between 3–5 Hz, which was confirmed by power spectrum analysis (data not shown).

**Fig. 11:**
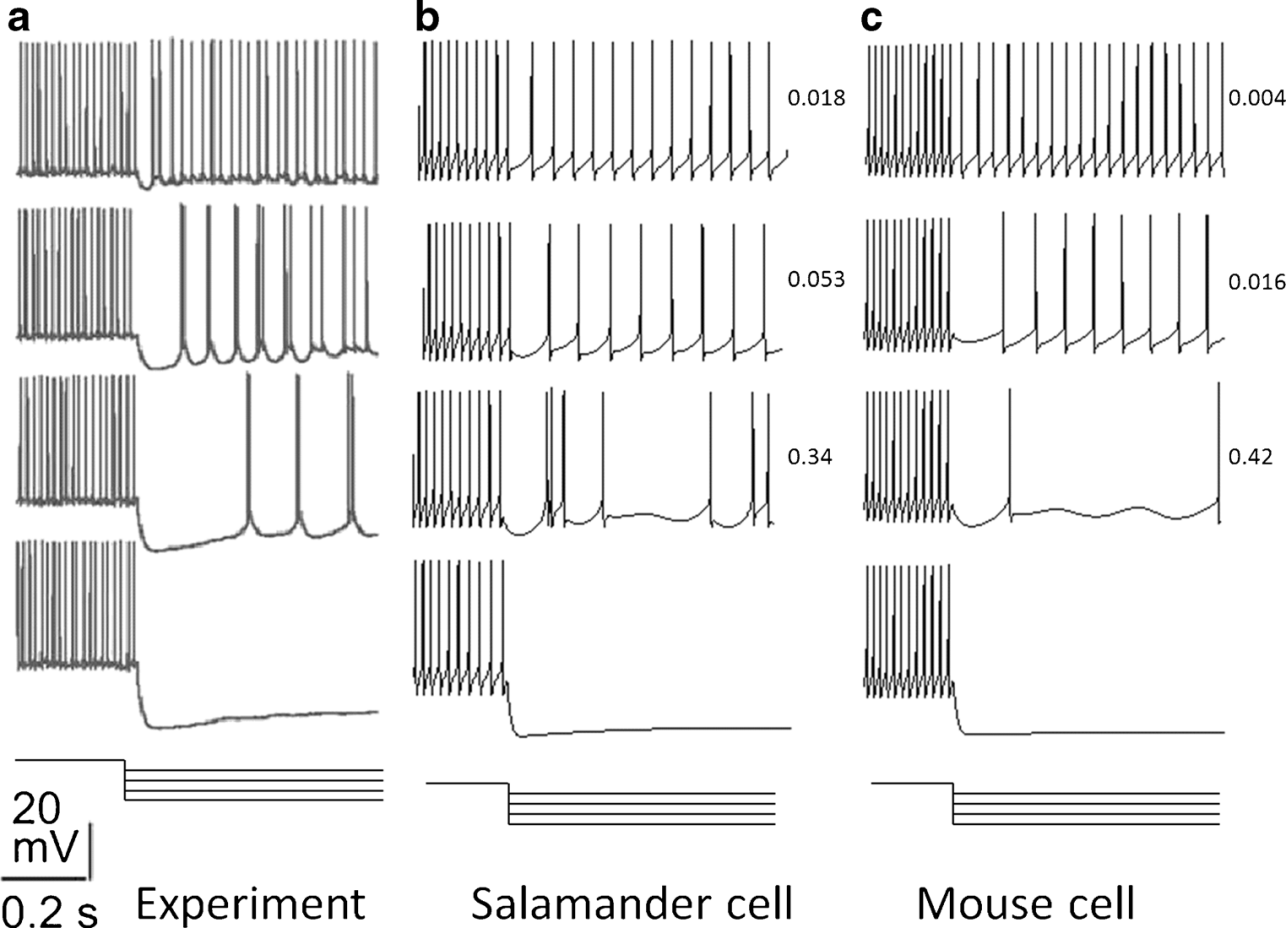
Comparison of the experimental results and simulated results for OFF S RGCs to increasing levels of hyperpolarizing current injection. Experimental and simulated responses show transitions from regular spiking to quiescence (V2) and an increase in the CV (V3). Numbers next to simulated responses show the CV. CV was calculated over 10 s of simulated data. The time and voltage scale applies to all plots. Current steps are superimposed and shown below voltage recordings. **a** Experimental data from Margolis and Detwiler (2007). **b** Simulation of a salamander cell. In these simulations, *ḡ*_NaP_ = 3.33 × 10^−6^ S/cm^2^, *ḡ*_T_ = 5.36 × 10^−4^ S/cm^2^, and *ḡ*_h_ = 10^−5^ S/cm^2^. **c** Simulation of a mouse cell. In these simulations, *ḡ*_NaP_ = 2.33 × 10^−5^ S/cm^2^, *ḡ*_T_ = 2.7 × 10^−4^ S/cm^2^, and *ḡ*_h_ = 10^−11^ S/cm^2^. Current steps for both simulation are: −0.02, −0.04, −0.06, −0.14 nA.

Figure 11 shows a comparison of the experimental results and simulation results for OFF S RGCs. Figure 11 shows that with an increasing level of hyperpolarization, cells transit from regular spiking to spiking at a lower frequency, to irregular spiking, to burst spiking and then to quiescence, satisfying V1–V3. Values of the CV is shown beside each simulated figure. The increasing values show that there is an increase in the spike irregularity.

**Fig. 12:**
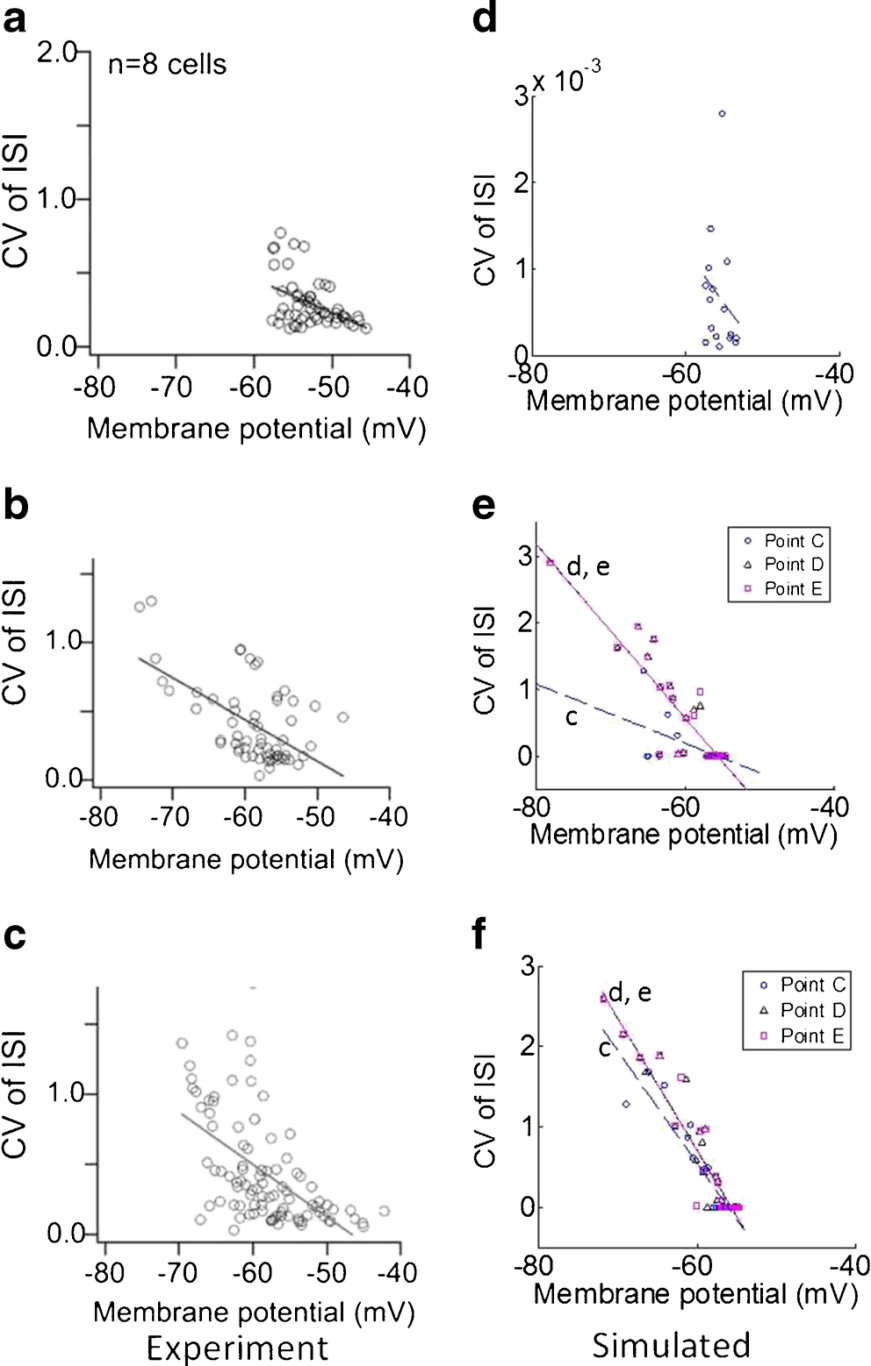
Comparison of the coefficient of variation of the interspike interval (CV of ISI) against membrane potential. **a**–**c** show experimental results from Margolis and Detwiler (2007). **d**–**f** show simulated results. **a** **d**, **b** **e**, and **c** **f** show results for ON, OFF T and OFF S cells respectively. Simulated results represent 10 cells that met constraints C0–C4. These cells were simulated with increasing negative current steps, to a maximum of −80 pA. Each marker in the OFF plots represent different conductance values for *ḡ*_T_, *ḡ*_NaP_, and *ḡ*_h_. A linear fit was applied to the data. In OFF plots, a letter indicates which fit it corresponds to. Note that the CV of ISI axis in simulated plots is different to the CV of ISI axis on experimental results. Experimental figures adapted from Margolis and Detwiler (2007) with permission

Figure 12 compares experimental values of the CV to simulated responses. Figure 12a–c are experimental responses for ON, OFF T and OFF S cells respectively. Figure 12d–e are simulated responses for ON, OFF T and OFF S cells respectively. In the simulated ON plot, conductance values corresponding to extreme conductances from the set in Fig. 8c were chosen. All conductance values produced similar results, therefore only the result for one conductance point is shown in Fig. 12d. In this plot, the conductance values are *ḡ*_NaP_ = 1 × 10^−5^ S/cm^2^, *ḡ*_T_ = 1 × 10^−5^ S/cm^2^, and *ḡ*_h_ = 1 × 10^−5^ S/cm^2^. In ON simulations, cells were first depolarized by injecting 50 pA. In the simulated OFF plots, 5 conductance points were taken from the OFF T and OFF S sets similar to the points A-E shown in Fig. 9. Since points A and B produced silent responses after small hyperpolarizing current steps, the results from these points were omitted from Fig. 12e–f. A linear fit was applied to the simulated data for points C-E. Note that the linear fit for the points D and E in the OFF T and OFF S sets are almost identical. We found that the slope of the fit is dependent on the conductance values. In particular, the slope of the fit became more negative as *ḡ*_T_ increased and *ḡ*_NaP_ decreased, however it had little dependence on *ḡ*_h_.

**Fig. 13:**
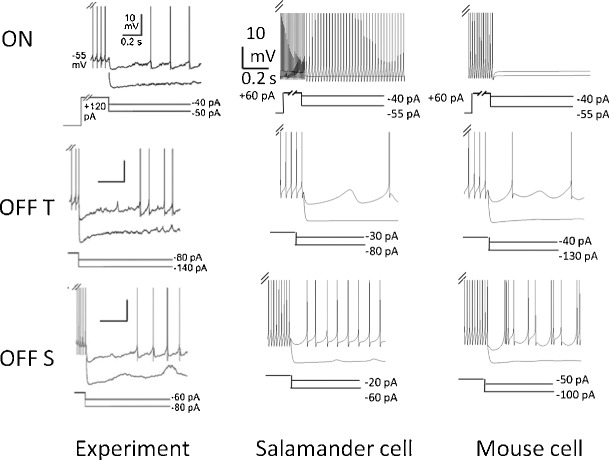
Cell transitions in response to an increasing amplitude hyperpolarizing current injection. Shown for comparison are two cells from the salamander and the mouse set, and experimental data (Margolis and Detwiler 2007). Cell responses are superimposed for different levels of current injection. Hyperpolarizing current produced different responses in different cells. Shown below each plot is the hyperpolarizing current levels used in the simulation. The same scale bar applies to all simulated plots. Salamander cell: In OFF S simulations, *ḡ*_NaP_ = 3.33 × 10^−6^ S/cm^2^, *ḡ*_T_ = 5.36 × 10^−4^ S/cm^2^ and *ḡ*_h_ = 10^−5^ S/cm^2^. In OFF T simulations, *ḡ*_NaP_ = 2 × 10^−6^ S/cm^2^, *ḡ*_T_ = 3.67 × 10^−4^ S/cm^2^ and *ḡ*_h_ = 10^−12^ S/cm^2^. Mouse cell: In OFF S simulations, *ḡ*_NaP_ = 6.67 × 10^−6^ S/cm^2^, *ḡ*_T_ = 4.69 × 10^−4^ S/cm^2^ and *ḡ*_h_ = 10^−4^ S/cm^2^. In OFF T simulations, *ḡ*_NaP_ = 10^−15^ S/cm^2^, *ḡ*_T_ = 4 × 10^−4^ S/cm^2^ and *ḡ*_h_ = 10^−6^ S/cm^2^. In both salamander and mouse ON simulations, *ḡ*_NaP_ = 10^−5^ S/cm^2^, *ḡ*_T_ = 10^−5^ S/cm^2^ and *ḡ*_h_ = 10^−5^ S/cm^2^. Experimental figures adapted from Margolis and Detwiler (2007) with permission

Figure 13 shows a comparison of the experimental results and simulation results for ON, OFF T, and OFF S RGCs from the mouse set. It can be seen that different hyperpolarizing current levels are required to generate oscillations in each cell. The level required to silence the output varied from cell to cell. In simulations, ON cells were first depolarized with a 60 pA current injection to elicit action potentials. ON cells showed no bursting at any stage. Bursting at intermediate levels of hyperpolarization in OFF T cells was a far less common feature than in OFF S. This is consistent with reports from Margolis and Detwiler (2007) where only 2 of 10 cells showed weak bursts. OFF S cells showed transitions from regular firing to irregular bursts, subthreshold oscillations and then to quiescence. This pattern in the transition was observed far less in OFF T cells. Our model showed that OFF T could only produce oscillations between bursts (see OFF T response in Fig. 13), whereas OFF S cells could produce sustained oscillations (see Figs. 9 and 10c). The amplitude of oscillations that were produced varied with conductance and morphology. In general, the amplitude of oscillations was 2–3 mV peak-to-peak; however, in some cases this reached around 10 mV.

**Fig. 14:**
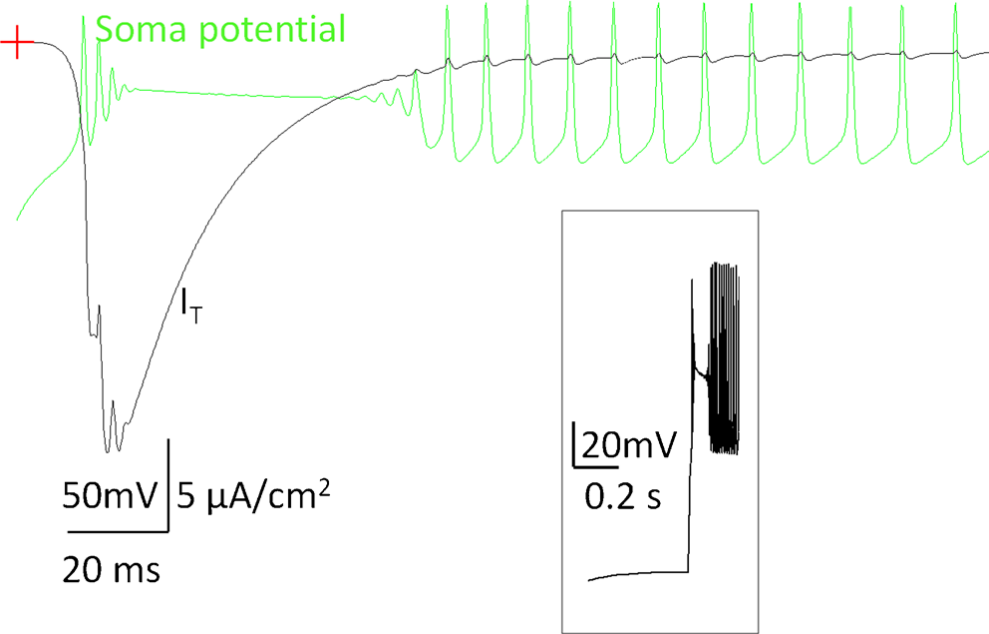
Latent period at the termination of a hyperpolarizing current step. Shown is the soma membrane potential (green trace) and *I*_T_ × 10^5^ (black trace) shown for comparison. Crosshair denotes the zero voltage point. The latent period is related to the inactivation time of *I*_T_ and can last anywhere between 20 and 80 ms. Insert shows a soma membrane potential on a smaller time scale for better visualization

We found that *ḡ*_T_ needed to be greater than 3 × 10^−5^ S/cm^2^ in order to generate high frequency bursting in OFF RGCs. Values of *ḡ*_T_ closer to the maximal level tended to produce a latent period where small oscillations were produced between bursts of action potentials. The duration of this latent period varied across cells but was directly correlated to the amount of *I*_T_ current induced by the termination of negative current injection and the inactivation time of this current to a lower amplitude. This phenomenon is not unusual for other cell types such as hippocampal neurons (Ullah and Schiff 2010). Figure 14 shows a soma membrane potential (green trace) and *I*_T_ current (black trace) on the same time scale. It can be observed that the latent period is related to the inactivation time of *I*_T_ current. In simulations, the latent period can last 20–80 ms.

In addition to the above, the level of hyperpolarization during a current step of −0.2 nA was recorded for all cells. The value of the membrane potential during hyperpolarization was 20–40 mV below the resting potential, which corresponds well with experimental data (Margolis and Detwiler 2007).

Also, the relative amplitude of the sag during a hyperpolarizing step of −0.2 nA was recorded for all cells. The amplitude was between 1 and 2 mV, which corresponds well with experimental data (O’Brien et al. 2002; Margolis and Detwiler 2007).

## Discussion

In this paper, we present realistic, morphologically correct models of 200 ON and OFF RGCs, and test ionic channel densities in morphological compartments that are necessary to capture experimentally recorded phenomena described by Margolis and Detwiler (2007). In particular, we showed the presence of subthreshold oscillations, burst firing, and spontaneous activity in the absence of synaptic input in OFF RGCs and the absence of such in ON RGCs. Our model predicted that the differences between ON and OFF cells are based on the presence of LVA Ca^2+^ current in OFF cells and absence of such in ON cells.

We found distinct sets of the parameters, *ḡ*_NaP_, *ḡ*_T_, and *ḡ*_h_, that correspond to ON and OFF cell populations. In general, the values in the ranges *ḡ*_NaP_ ≤ 10^−5^, *ḡ*_h_ ≤ 10^−5^, and *ḡ*_T_ ≤ 10^−5^ S/cm^2^ produced valid parameter sets for ON RGCs. The values in the ranges *ḡ*_NaP_ ≤ 3 ×10^−5^, *ḡ*_T_ ∈ [3 × 10^−5^, 4 × 10^−4^], and *ḡ*_h_ ≤ ×10^−5^ S/cm^2^ produced valid OFF cell parameter sets. These values were similar to those found in single compartment models described by Kameneva et al. (2011). The present study, however, extends the results of previous modeling studies by explicitly examining the effect of changes to the single compartmental morphological structure of the neurons.

This study shows that not all cell morphologies match the RCG behavior described by Margolis and Detwiler (2007). We found that the cells that were able to reproduce the described phenomena had smaller total surface area, *S*_total_, and in general, a smaller ratio of dendritic to total surface area, *R*_dend,total_. This illustrates that morphology plays an important role in shaping the cell’s response. These results are in agreement with Fohlmeister and Miller (1997), who showed that the cell morphology play a role in firing patterns and the impulse frequency response. However, Fohlmeister and Miller (1997) also claimed that the ion channel distribution plays a secondary role in these functions. However, our results illustrate how the channel density distribution plays an important role in intrinsic electrophysiological properties of RGCs.

Since the NeuroMorpho database does not distinguish the cell subtypes, or whether they are ON or OFF RGCs, it is unclear what morphological cell types were actually able to satisfy the modeling constraints. Determining the subtypes of the 200 cells used in this study requires electrophysiological identification on each cell model. This investigation is left for future work.

The difference in LVA Ca^2+^ current in ON and OFF RGCs suggests that this current contributes to differences in the way these cell types process visual information. It was proposed that LVA Ca^2+^ current initiates rebound excitation in OFF RGCs to generate a precisely timed OFF response at the termination of a light stimulus (Margolis et al. 2010). Activation of LVA Ca^2+^ current provides a mechanism that couples light offset to a precisely timed burst of spikes. At the onset of light, OFF RGCs receive mostly inhibitory synaptic input from bipolar cells. Inward currents in dendrites (such as calcium currents) allow OFF cells to convert local inhibitory synaptic potential into an excitatory signal that robustly reaches the soma. ON cells, on the other hand, do not require such a mechanism to signal the light onset, which is triggered mostly by excitatory synaptic input.

Functional implications of LVA Ca^2+^ current signaling in other types of neurons are the focus of ongoing investigation (Destexhe et al. 1998). Dendritic LVA Ca^2+^ current plays an important role in the efficient modulation of thalamic burst discharges by corticothalamic feedback. Dendritic LVA Ca^2+^ current may explain differences in the intrinsic firing properties between thalamic reticular neurons recorded *in-vitro* and *in-vivo* (Destexhe et al. 1998).

It is important to examine the properties of the high density sodium channel band (SOCB) of the axon that was found in RGCs by Carras et al. (1992) and Fried et al. (2009), and others. It was shown previously that, in RGCs, the SOCB has the lowest threshold for electrical stimulation (Fried et al. 2009), and the effect of SOCB geometry and density on the cell’s response to electrical stimulation was investigated computationally by Jeng et al. (2011). In some studies, the density of sodium channels was increased by 50 times over that of the soma in order to reproduce experimental results for pyramidal neurons (Kole et al. 2008).

In our modeling, the SOCB produced a low threshold region for action potential initiation. The absence of this higher density region (*ḡ*_Na_ *R*_SOCB,soma_ = 1) resulted in the low threshold region varying from cell to cell. In some cases, the low threshold region was present in dendritic compartments of the cell. This had the effect of producing unphysiological behavior in the soma and could not ensure orthodromic propagation of action potentials. Increasing the *ḡ*_Na_ *R*_SOCB,soma_ ensured that the low threshold region was always in the high density sodium region of the axon.

While our modeling was based on mice RGCs, it has been shown that intrinsic membrane currents in cells of primates are similar to those in lower vertebrates (Han et al. 2000). Also, our modeling did not distinguish between ionic channel subtypes. For example, it has been found that Na_v_1.6 and Na_v_1.1 are present in RGCs (Caldwell et al. 2000). However, how different activation thresholds of channel subtypes affects spiking properties of the cell was not investigated here. On the other hand, passive properties of the cell also shape action potential generation. In our models, all cells had the same axon geometry. How the length and diameter of axon affects action potentials is left for future studies.

This study sought to understand the effect of cell morphology on the mechanisms underlying differences between ON and OFF RGCs. An understanding of the mechanisms generating phenomena that are different for ON and OFF RGCs can assist in developing successful stimulation strategies for retinal implant devices. The models of ON and OFF RGCs developed here can be used to examine the possibility of differential stimulation of these cell classes by a visual prosthetic device. Since it was shown that ganglion cells are stable after degeneration-induced change in synaptic input (Margolis et al. 2010), our model may be adequate for describing this situation. Multicompartment models of RGCs may be used to investigate the effect of the stimulation electrode position on the threshold for firing, which is a crucial question for stimulation strategies for a successful visual implant.

How effectively synaptic and regenerative potentials propagate within neurons depends critically on the membrane properties and intracellular resistivity of the dendritic tree (Stuart and Spruston 1998). These properties can be examined using models such as ones described here by adding synaptic input. Also, it was shown that the noise in the dendrites has a large effect on the spike precision (van Rossum et al. 2003). Also, the bursting patterns vary substantially in the presence of noise (Channell et al. 2009). Our model can be extended to explore underlying mechanisms of these phenomena.

Dendritic shrinkage study of RGCs in cats with glaucoma showed that the cell soma size, total dendritic length, and number of branch bifurcations of dendrites decreased significantly in glaucomatous eyes compared with normal ones (Shou et al. 2003). How these changes in morphology affect the cell’s capacity to fire action potentials can be investigated using multicompartment models such as those developed in this study.
